# Mathematical Modeling of Tuberculosis Bacillary Counts and Cellular Populations in the Organs of Infected Mice

**DOI:** 10.1371/journal.pone.0012985

**Published:** 2010-09-23

**Authors:** Antonio Bru, Pere-Joan Cardona

**Affiliations:** 1 Departamento de Matemática Aplicada, Facultad de Matemáticas, Universidad Complutense de Madrid, Madrid, Spain; 2 Unitat de Tuberculosi Experimental, Fundació Institut per a la Investigació en Ciències de la Salut Germans Trias i Pujol, Universitat Autònoma de Barcelona, Badalona, Spain; 3 Centro de Investigación Biomédica en Red (CIBER) Enfermedades Respiratorias, Instituto Carlos III, Palma de Mallorca, Spain; McGill University, Canada

## Abstract

**Background:**

*Mycobacterium tuberculosis* is a particularly aggressive microorganism and the host's defense is based on the induction of cellular immunity, in which the creation of a granulomatous structure has an important role.

**Methodology:**

We present here a new 2D cellular automata model based on the concept of a multifunctional process that includes key factors such as the chemokine attraction of the cells; the role of innate immunity triggered by natural killers; the presence of neutrophils; apoptosis and necrosis of infected macrophages; the removal of dead cells by macrophages, which induces the production of foamy macrophages (FMs); the life cycle of the bacilli as a determinant for the evolution of infected macrophages; and the immune response.

**Results:**

The results obtained after the inclusion of two degrees of tolerance to the inflammatory response triggered by the infection shows that the model can cover a wide spectrum, ranging from highly-tolerant (i.e. mice) to poorly-tolerant hosts (i.e. mini-pigs or humans).

**Conclusions:**

This model suggest that stopping bacillary growth at the onset of the infection might be difficult and the important role played by FMs in bacillary drainage in poorly-tolerant hosts together with apoptosis and innate lymphocytes. It also shows the poor ability of the cellular immunity to control the infection, provides a clear protective character to the granuloma, due its ability to attract a sufficient number of cells, and explains why an already infected host can be constantly reinfected.

## Introduction

*Mycobacterium tuberculosis* is the most insidious microbial human pathogen known. This bacillus is able to induce an infection in the human body, the so-called latent tuberculosis infection (LTBI), which can persist for a long period of time, even, according to some authors, for the whole life[1]. It is currently considered that around a third of the world's population (2.5 billion people) has an LTBI. Some 10% of these people will go on to develop active TB[2], thus accounting for the 9 million new cases and 2 million deaths every year [3]. This is a catastrophic process with no apparent end in sight as all these TB infections constantly generate new LTBI cases (approximately 100 million a year) [3].

The life cycle of the bacilli "in vitro" and its interaction with the alveolar macrophages, including how it interacts with the cytoplasm organelles and how these cells interact with the body as a whole by inducing the secretion of cytokines, is currently well-understood [4]. Likewise, the antigenic content of the bacilli, and which antigens are able to induce a protective immune response, is also well known[5]. Unfortunately, the scenario where all these entities are thought to interact, namely the granuloma, is less well understood. Some information regarding the evolution of the granulomas has been obtained from necropsies, and several phases in its development process have been identified; in particular, its induction process has been mimicked "ex vivo"[6] and filmed "in vivo" in the liver[7]. All these findings provide us with different views of the host-pathogen interplay, although they occur on different spatial and temporal scales and are therefore often very difficult to bring together. The use of systems biology, which implies the construction of computational models that follow a detailed sequence of rules implemented directly in object-orientated programming languages, therefore appeared to us to be a useful tool to try to structure all this knowledge and gain a better understand of the onset of granuloma formation in *M. tuberculosis* infection[8].

The first intensive studies to evaluate the evolution of these lesions were performed by Kischner et al., who attempted to simulate the induction of solid and necrotic granulomas, corresponding to protective and deleterious responses respectively, in macaques infected with *M. tuberculosis* [9]. These authors used the cellular automata system, which is based on a 2D lattice-based model where individual cells evolve on the basis of temporal and spatial rules which are mainly at random depending on a probability. This system requires that the initial states of each cell are first specified, along with the instructions that the computer will follow to determine their state in the next time step. These instructions typically depend on the states of the surrounding cells, and a new step or generation is created each time the rules are applied to the whole grid [8]. Use of this technique, along with the integration experimental data obtained from different systems, has allowed us to address the question of how granulomas are induced, the role of the immune response in their formation and how their formation influences the bacillary growth.

The natural history of LTBI starts with inhalation of an infected aerosol, which allows the bacilli to reach the alveolar spaces and subsequently to be phagocytosed by the alveolar macrophages. The bacilli avoid the phagolysosome union[10], [11], [12] and grow until they destroy the macrophage, which in most cases results in necrosis if the macrophage has not previously induced its own apoptosis[13]. This rupture of the infected macrophage temporarily stops bacillary growth by releasing them into the stressful extracellular milieu[14], [15], [16], where it is thought that cannot grow [17], [18]. These bacilli can potentially remain embedded in the necrotized environment for a long period of time until they are phagocytosed by another macrophage.

The presence of natural killers (NK)[19], plays a relevant role at this stage as they can activate macrophages and cause a small amount of bacillary destruction[20]. In this sense, the anti-inflammatory role that has recently been assigned to the presence of neutrophils[21], which were previously thought to have a bactericidal effect when apoptotic[22], [23], should be noted. Likewise, neutrophil necrosis may also occur in the extracellular matrix, thereby curtailing bacterial dissemination[24] and contributing to the formation of a granulomatous structure that can support sudden cellular entrance[25].

In this scenario, approximately 10% of monocytes[26], [27] become dendritic cells (DCs)[28], which, once infected, migrate towards the regional lymph nodes where they present *M. tuberculosis* antigens and induce the proliferation of specific T lymphocytes[29], [30]. These lymphocytes, mainly type I CD4 with some type I CD8[4], [31], then migrate towards the infection site, where they recognize the infected macrophages and activate them by IFN-γ or cause their death by necrosis or apoptosis, thus controlling the bacillary load[29], [30]. This process is faster for immune hosts as they already have memory T lymphocytes, thus allowing a faster generation of effector T cells[32]. Another process, namely the removal of cellular debris by macrophages, which progressively become filled with lipid bodies to become foamy macrophages (FMs), takes place simultaneously[33].

The slow pace of bacillary growth results in a discrete pathological process at the beginning of the infection. Thus, in an experimental murine model, where infection is induced with a low-dose aerosol, infected lungs show a very limited and transient localised increase in the cellularity between the epithelia and the lamina propria rather than granulomatous lesions in the first three weeks post-infection despite the fact that the bacillary load increases up to 10^5^ CFU[34], [35]. Granuloma formation depends entirely on TNF production by the infected macrophages and T cells, as well as the integrity of all ligands and receptors of the TNF family. Sustained TNF signaling is required to maintain the necessary local chemokine gradients to hold the cells in close apposition, thus favoring the activation of infected macrophages[36], [37], [38], [39].

The bacillary life-cycle status[40], [41], [42], [43] also undergoes various transformations according to changes in the environment. Thus, the bacilli undergo an exponential growth, the *log* phase, inside the phagosomes of the macrophages[44], a stationary phase, which starts when the bacilli are exposed to the stressful extracellular milieu or when they are located inside the phagolysosomes of activated macrophages[16], [45], [46], and the *lag* phase, which occurs once these bacilli return to the phagosomes until they restart the bacillary growth. The length of the *lag* phase increases proportionally to the time of the previous stationary phase (i.e. submitted under stress)[47], [48]. Once the *lag* phase is over the early *log*-phase, when the division process starts but there is as yet no real division, starts[49]. In fact, it is traditionally accepted that those bacilli that reach this stationary state can remain inside old lesions for long periods, even for the lifetime of the host, where they are responsible for generating the LTBI and can reactivate to induce active TB under the influence of resuscitation factors and one or more immunosuppressive factors[50]. There is growing evidence that supports the idea of LTBI occuring as a result of a constant endogenous reinfection of the host, the so-called dynamic hypothesis[51], and active TB as the result of an episode of reinfection in the upper pulmonary lobes, where the high oxygen pressure favors faster bacillary growth. This assumption is based on the constant drainage of non-replicating bacilli towards the alveolar space via the FMs, which are incorporated into the aerosols generated with the inhaled air upon reaching the alveolar fluid[52] and can therefore be transferred into the pulmonary parenchyma and generate new lesions[51].

By using a cellular automata system, the aim of the current study is to bring all these concepts together in a rational manner by creating a model that can explain the dynamics of the onset of granuloma formation. This model is based on a murine experimental model as this is the "in vivo" model for which most information is available. Furthermore, the addition of data obtained from *ex vivo* and *in vivo* assays in mice, guinea pigs and mini-pigs should provide a better understanding of the main parameters that induce the formation of granulomas, the importance of this process in control of the infection, and indicate how this progression occurs in humans.

## Results

### Validation of the model

Two criteria were followed to validate the model and fit it to the experimental data, namely progression of the bacillary load and granuloma size. The “in vivo” data used to validate the model were obtained from studies involving the low-dose (i.e. about 50 CFU) aerosol infection of immunocompetent (i.e. C57BL/6) and highly immunosuppressed mice, the latter of which lack specific lymphocytes (i.e. SCID mice). Both were infected with the virulent H37Rv strain of *M. tuberculosis*, for which a lot of data is available[35], [53]. We considered this experimental murine model to be “highly tolerant” (HT) to infection due to its ability to harbour a large bacillary concentration without hampering its health status[54], [55]. We also developed a “poorly tolerant” (PT) model on the basis of data obtained in a “human-like” experimental model, namely mini-pigs, after inoculating 10^3^ CFU transthoracically[56]. Modeling was performed starting with the inoculation of 25 CFUs into the 2D lattice and considering the onset of the immune response (on the basis of the entrance of specific lymphocytes, Ts) approximately two weeks after the challenge (10,000 iterations).

#### Tolerance, as defined by the chemokine threshold (CT) needed to attract a cell, was used to determine the ability to control the bacillary load

**Figure 1:**
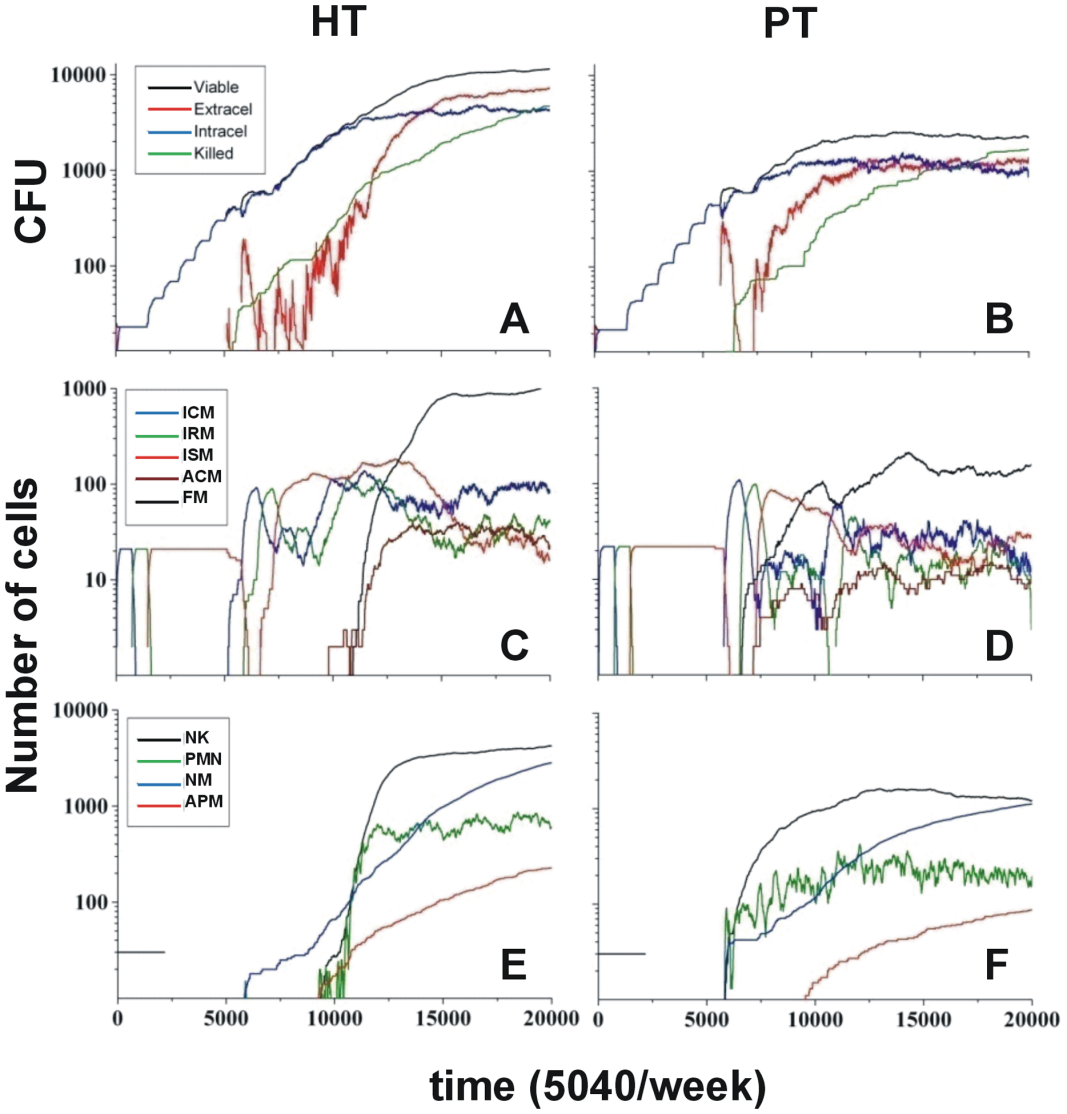
Evolution of the infection without an adaptative immune response. Evolution of the infection without inducing an adaptative immune response in highly (HT) and poorly tolerant (PT) hosts according to the chemokine threshold above which a new cell can be attracted to a neighboring square (defined as 1000 arbitrary units (a.u.) and 650 a.u., respectively). A and B show the evolution of the bacillary load, and C and D the evolution of the different kind of macrophages. E and F show the evolution of natural killers (NK), neutrophils (PMN), and necrotic (NM) and apoptotic macrophages (APM).

**Figure 2:**
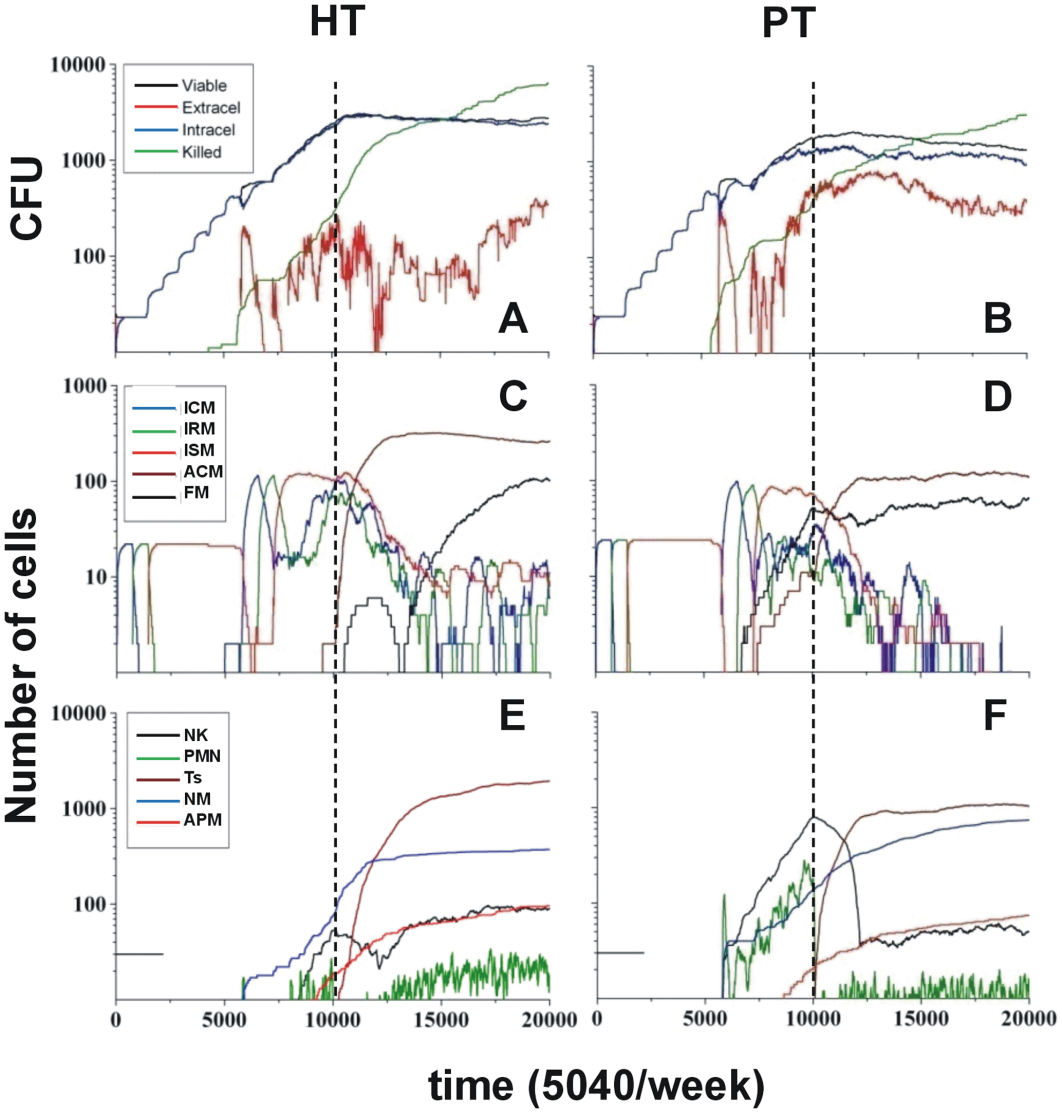
Evolution of the infection with the presence of an adaptative immune response. Evolution of the infection, including the presence of an adaptive immune response, in highly (HT) and poorly tolerant (PT) hosts according to the chemokine threshold above which a new cell can be attracted to a neighboring square (defined as 1000 arbitrary units (a.u.) and 650 a.u., respectively). A and B show the evolution of the bacillary load, and C and D the evolution of the different kind of macrophages. E and F show the evolution of natural killers (NK), neutrophils (PMN), and necrotic (NM) and apoptotic macrophages (APM). Appearance of the immune response is marked with a dotted line.

The evolution of the viable bacilli in both scenarios (with or without adaptive immune response; see Figures 1A and 2A) showed that the bacillary load in the HT host reached 3×10^3^ and 1×10^4^ CFU respectively. Taking into account that these values were obtained for a 2D scenario, we extrapolated an approximate 3D scenario by multiplying these values by ten, in other words by superimposing 10 2D lattices, to give values of 3×10^4^ and 1×10^5^ CFU respectively, which are in the range of those observed experimentally[35], [53]. This scenario was obtained with a CT of 1000 a.u. (arbitrary units).

To find the CT for a PT host, we assayed lower values, starting with a CT of 500 a.u. We did not obtain any difference between the predicted values with and without immune response at this level, a fact that does not fit with the current experimental and clinical data, therefore we gradually increased the CT value until we obtained a twofold reduction of the bacillary load with an immune response at CT = 650 a.u. (Figures 1B and 2B). Furthermore, this response was able to sterilize the lesion, whereas the non-immune model was only able to maintain the chronic infection.

#### Initial bacillary growth is independent of the tolerance level, although this parameter finally defines the bacillary control

Evolution of the infection was marked by an initial period of time with no bacillary growth, as the bacilli were in T*lag* and Tearly-*log* phases inside infected-consolidated (ICMs) and infected-at risk macrophages (IRMs; Figures 1 and 2, C and D) in both hosts, irrespective of their immune status. A “step-like” growth evolution (five steps) that reflected the increase in bacillary load corresponding to the duplication time inside an infected-sentenced macrophage (ISMs), then appeared. The differences between both progressions became apparent at the end of this period (Figures 1 and 2, A and B). The bacilli were also at risk being killed upon release into the extracellular milieu once the ISM become apoptotic (APM). However, most of them were phagocytosed, thus infecting resting macrophages (RMs), after release from necrotic macrophages (NMs; Figures 1 and 2, A and B). The main difference between the HT and PT hosts was found in the capacity to react against the high concentration of chemokines released by necrotic macrophages. Thus, NK and polymorphonuclear neutrophils (PMNs) appeared in the PT model at this point, whereas they were absent in the HT host (Figures 1 and 2, E and F). This induced the production of activated macrophages (ACMs), which required 4000 more iterations (i.e. 6 days) to occur in the HT model (Figures 1 and 2, C and D).

#### Strong innate immunity in PT hosts was key to control of the bacillary load but also increases the amount of necrotic macrophages and extracellular bacilli

Control of the bacillary load depended on both the ability to necrotise macrophages and the ability to produce a sufficient number of activated macrophages (ACMs) to prevent the bacilli from growing intracellularly, either by killing them or leaving them in a non-replicating state (*Bnr*). Furthermore, bacillary destruction was initiated first in the HT host due the presence of apoptotic macrophages (APMs), which meant that extracellular bacilli could be killed by any non-activated macrophage (Figures 1 and 2, A and B), although this did not preclude a better control. The lack of activation of infected macrophages because of the lack of NK resulted in an increase in the bacillary load in this scenario.

The number of extracellular bacilli subsequently increased more quickly in the PT host (Figures 1 and 2, A and B) as a result of the activation process, which leads to both macrophage activation and the induction of necrosis in the infected macrophages. This process also results in the release of extracellular bacilli, which was clearly higher in the PT host (Figures 1 and 2, E and F).

This initial increase in NMs, PMNs and NK also induced a quicker increase in the concentration of foamy macrophages (FMs) in the PT host (Figures 1 and 2, C and D) despite the fact that these cells were drained towards the alveolar space, thus also contributing to a decrease in the bacillary load in the lattice. Indeed, up to 750 FMs, carrying up to 1600 bacilli (Figure S1), were drained off, thus contributing significantly to the bacillary drainage. A similar increase in NK and PMNs, occurred later in the HT host, together with an increase in FMs as these cells were not drained in this case.

#### The adaptive immune response dramatically affects the bacillary load in HT hosts

The entrance of specific T lymphocytes (Ts) was clearly related to the appearance of activated macrophages (ACMs) and increased the rate of bacillary killing in the HT host, whereas in the PT host it increased the presence of ACMs but did not affect that much the rate of bacillary destruction (Figure 2, A, B, C and D). The number of killed bacilli was higher than the number of viable bacilli and the number of extracellular bacilli was lower than the number of intracellular bacilli in both cases, which is in contrast to the situation found over the same period (10,000 to 20,000 iterations, i.e. from 2 to 4 weeks) in the absence of adaptive immunity. This effect was especially dramatic in the HT host, which maintained a lower extracellular bacilli count than the PT host up until the end of the study period, when both were similar (Figure 2 A and B). Likewise, the number of newly infected macrophages decreased in both cases, thus reflecting a clear control of the infection process. The decrease of PMNs was notable in both cases as this was included in the model premises, therefore the number of drained FMs in the PT host was also reduced, i.e. 88 FM, carrying up to 300 bacilli (Figure S1).

### The tolerance level affects granuloma formation and the onset of bacillary control

**Figure 3:**
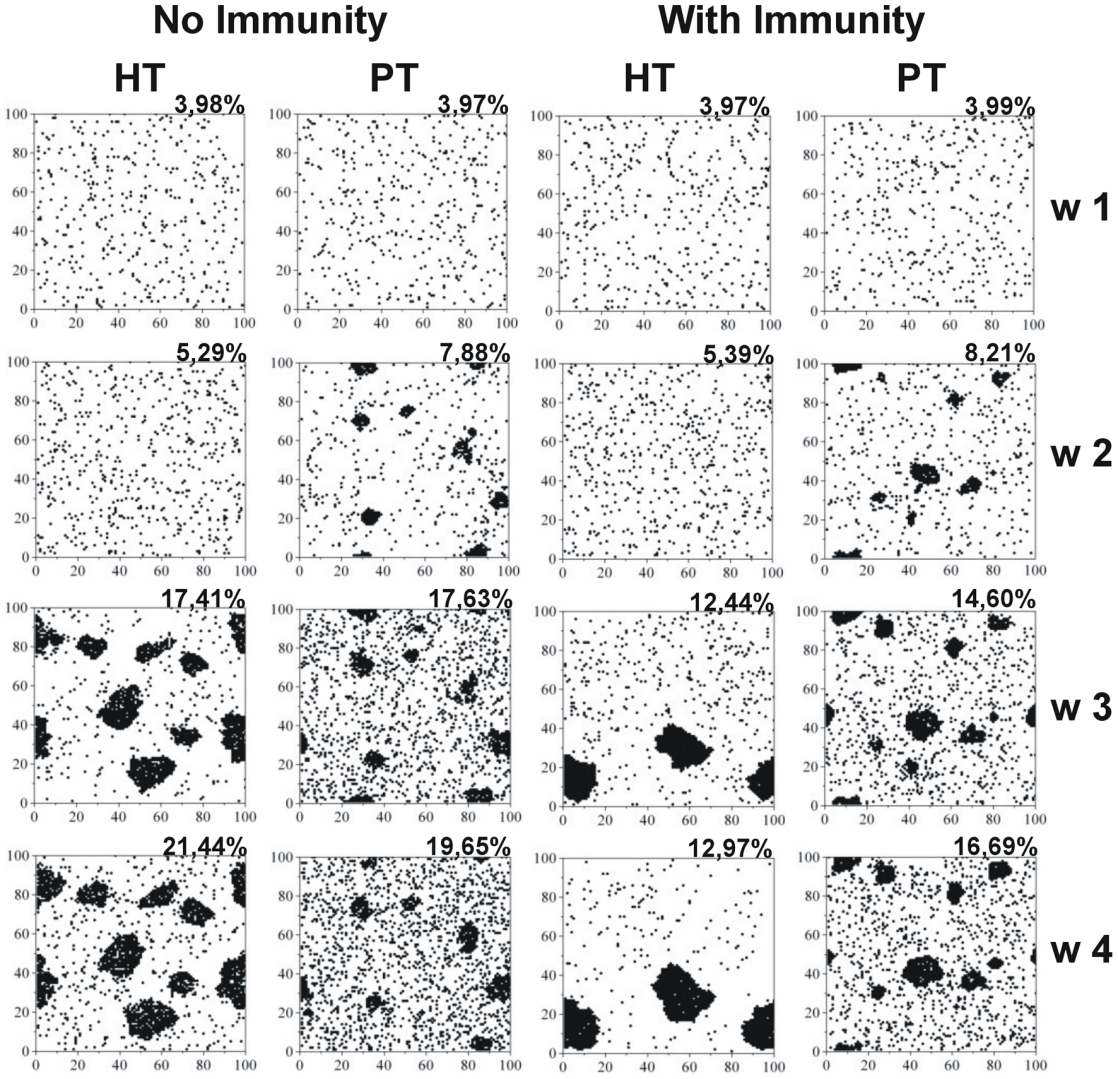
Cellular infiltration. Evolution of the cellular infiltration caused by viable macrophages in the four cases analyzed in terms of the host's tolerance and immunological status. Squares represent the whole lattice assayed, and show the evolution every week. Percentages show the ratio between the number of cells occupied by a viable macrophage divided by the total number of cells.

Progression of the granuloma size was the second parameter used to validate the current model. Figure 3 shows the evolution of the granulomas in the four models obtained. The results obtained in the HT host show that granulomas began to appear from week 3 post-infection. Interestingly enough, observation of the whole course of granuloma evolution (videos S1 to S4 in supplementary material) showed the formation of small granulomas prior to week 3, although they rapidly disappeared. Figure S2 shows pictures of lung granulomas obtained at weeks 3 and 4 post-infection in C57BL/6 mice and in mini-pigs (week 5). It can be seen from this figure that the granulomas have a diameter of about 0.4 mm, just as predicted by the model. The onset of adaptive immunity apparently reduces the number of granulomas, and also the global degree of infiltration, although it tends to increase the size of each particular granuloma. The evolution of granulomas in the PT host appeared to be significantly different, as they appeared before the onset of adaptive immunity.

**Figure 4:**
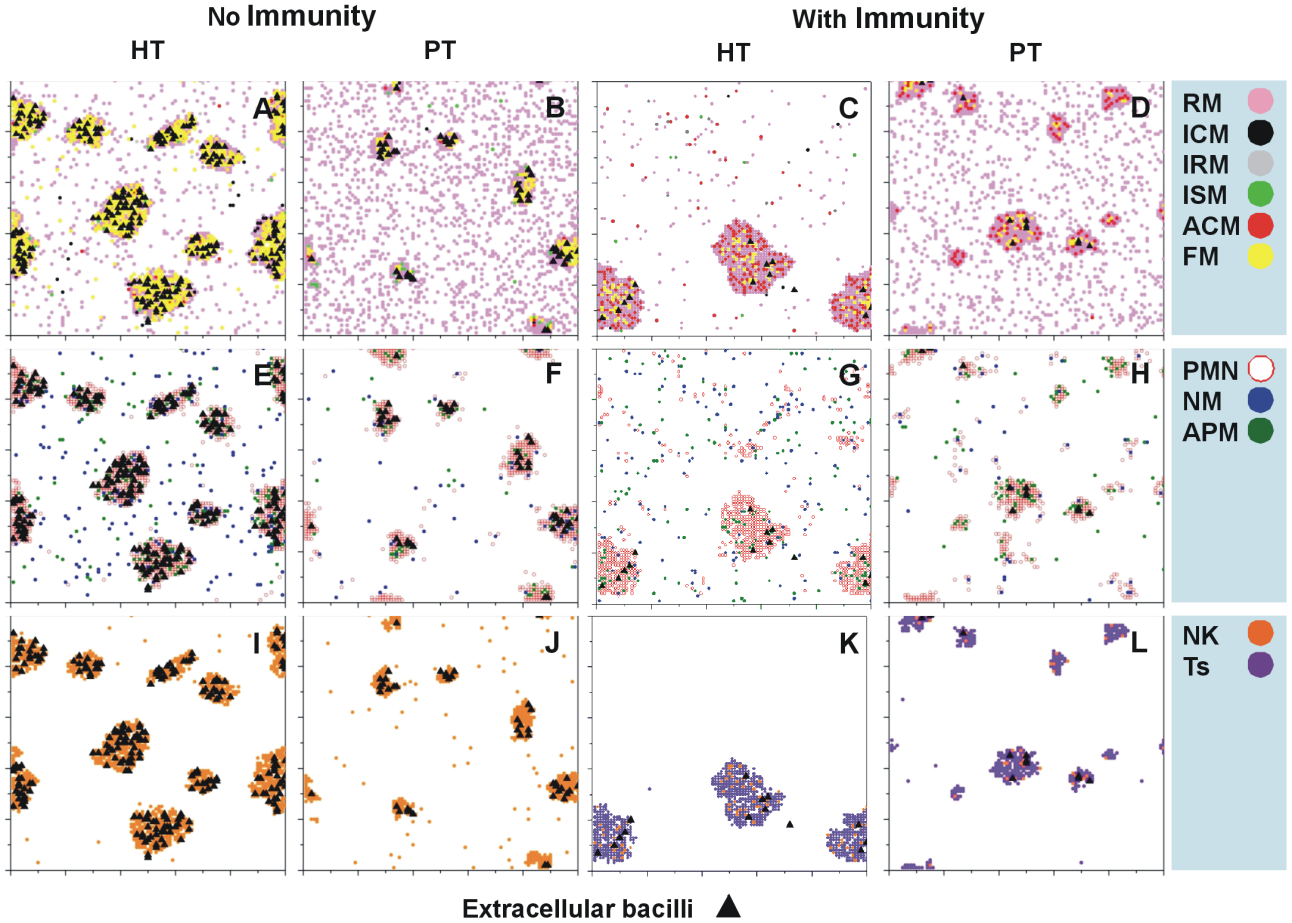
Characteristics of the granulomas. Characteristics of the granulomas taking into account the majority of the cells at week 4 post-infection in the four cases studied in terms of the tolerance and immune status. Squares represent the whole lattice assayed.

A more detailed view of the granuloma formation at the end of the evolution (i.e., 20,000 iterations, or 4 weeks post-infection) is given in Figure 4, which shows a clear predominance of FM cells in the HT model with no immune response, even outside the granuloma. In contrast, this predominance is much less evident in the PT thanks to the drainage of these cells.

Remarkably, a high number of necrotized macrophages are also present in the lattice, with a large number of these being found outside the granuloma. A high accumulation of activated macrophages is clearly seen in the models with adaptive immunity, even outside the granuloma in the HT hosts. Furthermore, a high number of infected macrophages, which are non-activated and dispersed throughout the parenchyma, are found outside the granuloma in the models without adaptive immunity and even in HT hosts with adaptive immunity. The presence of resting macrophages (RMs) throughout the parenchyma in the PT hosts, which decreased in number once adaptive immunity appeared as they accumulated on the already existent granulomas, is also of importance. This finding is related to their ability to stimulate the presence of new cells with lower chemokine concentration.

### Chemokine levels and distribution determine the formation of granulomas

**Figure 5:**
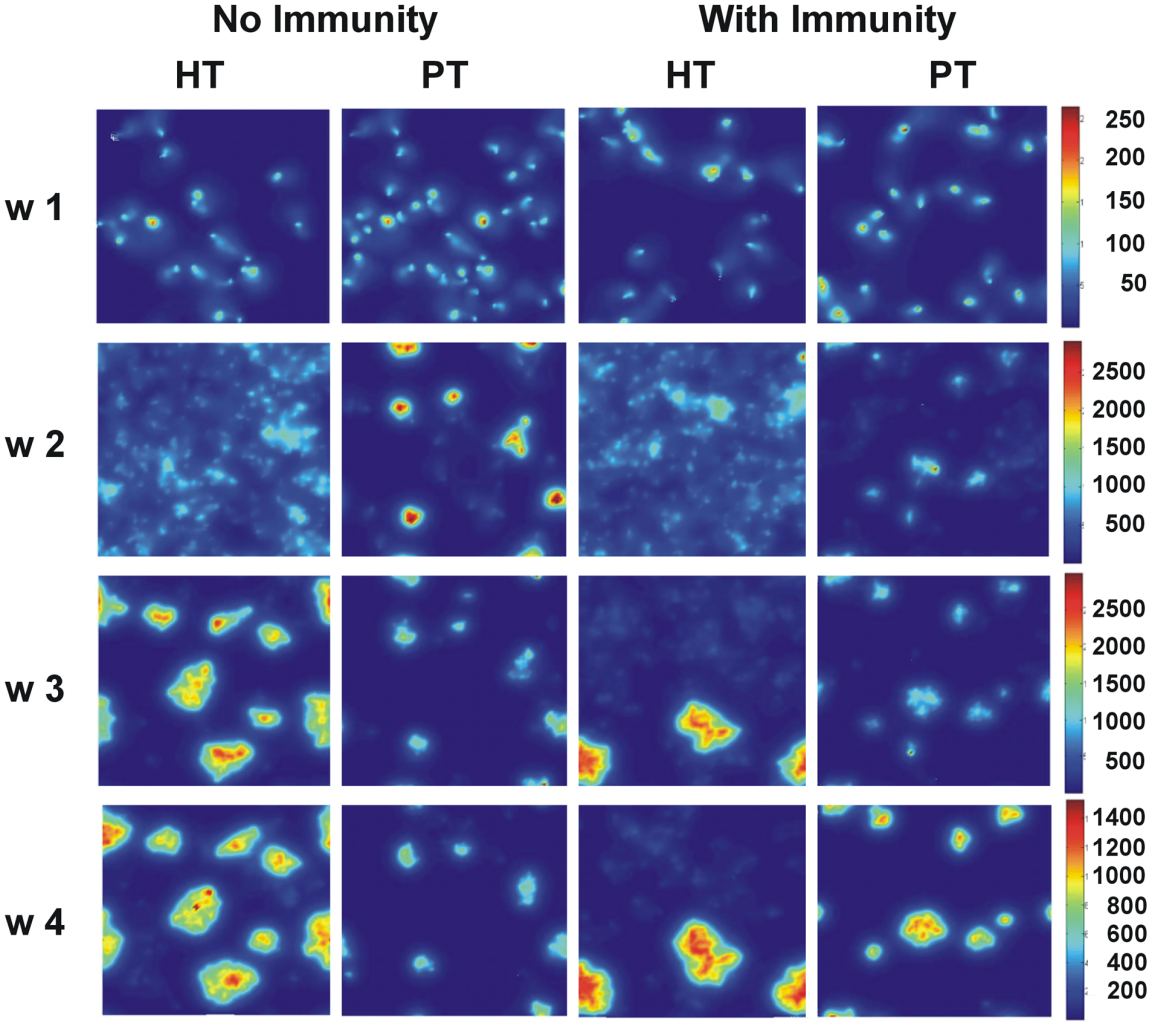
Chemokine concentration. Evolution of the chemokine concentration in the four cases analyzed in terms of the host's tolerance and immunological status. Squares represent the whole lattice assayed, and show the evolution every week.

Figures 5, S3, s4, together with the S1 to S4 videos provided as supplementary material, clearly show that a strong and continuous secretion of chemokines is required for granuloma formation. Figure S4, which shows the evolution of total chemokines accumulated in every lattice, perhaps best illustrates this point. Thus, it shows how the PT host, with a lower level of chemokines, is able to induce a better bacillary control, whereas the HT host needs a higher level. Indeed, it is interesting to note that the higher chemokine concentration in the latter host does not signify better control in the absence of an adaptive immune response.

Taken together, the above findings suggest that the individual production of chemokines is not sufficient for granuloma formation and that the random aggregation of cells with the capacity to excrete chemokines controls granuloma formation.

### A higher apoptosis rate significantly increases the ability to control bacillary growth

Figure S5 clearly shows that a 10-fold increase in the probability of inducing apoptosis notably decreased the bacillary load in all cases, even in the PT model with an immune response. The critical point appears at the fifth bacillary duplication cycle when, according to our modeling process, there is a greater probability of apoptosis occurring.

### Onset of the immune response does not alter the initial intracellular bacillary growth

Previous experimental evidence (see Figure S6) has linked onset of the immune response with the appearance of infected dendritic cells (DCs) in the regional lymph nodes[29], [30]. Thus, the initial increase in DC formation after necrosis of infected macrophages is caused by the increase in the number of extracellular bacilli, therefore a sudden increase in DCs could potentially trigger the adaptive immunity. As expected, the PT host experimented a sudden higher increase at 6000 iterations, after which there was a constantly increasing trend that led to all the models converging at around 10,000 iterations. The HT hosts increased at a faster rate after 6000 iterations to balance the initial lower concentration.

**Figure 6:**
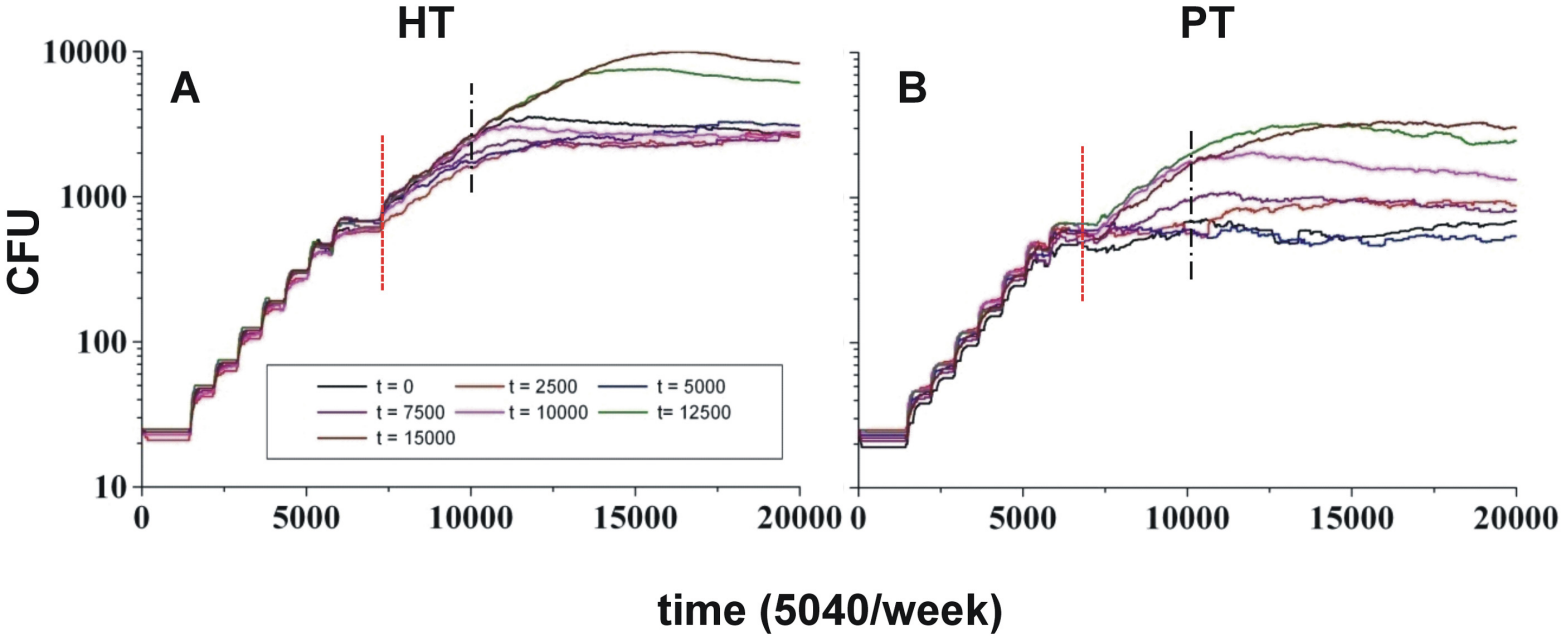
Availability time for specific T lymphocytes. Effect of the availability time for specific T lymphocytes for HT and PT hosts (pictures A and B). Appearance of the change in the evolution of CFU counts is marked by a red dotted line; while the beginning of the usual immune response (10000 time-steps or iterations) is marked with a black hashed line.

Figure 6 shows how the onset of adaptive immunity did not affect the bacillary growth observed at the beginning of the infection, even at time 0, thus reflecting the establishment of new infectious foci in a host with effector cells already circulating.

This is followed by a period, at between 7000 and 10,000 iterations, that appears to result in some changes to the bacillary load, especially in the PT host, which is clearly more sensitive to the onset of the immune response as it react more quickly to the chemokine level. As the HT host needs higher chemokine concentrations (as shown in Figure S4), it displays two patterns of reactivity: when the Ts presence starts in the later stages (12,500 iterations) there is a clear decrease in bacillary control, whereas in the other cases no differences were seen.

### Lag period influences the bacillary load

Figure S7 shows the distribution of the bacillary T*lag* periods for the four models. This period increases with time in the HT host and also displays a higher variability, thus reflecting the presence of bacilli that tend to accumulate in the extracellular milieu, thereby increasing the T*lag* values. In contrast, the PT host tends to maintain low values, with a high turnover between the intra- and extracellular bacilli. This could be related to the larger size of the granulomas in the HT host, which would make the extracellular bacilli less accessible to the macrophages due to the higher number of necrotised macrophages. In both cases, the onset of adaptive immunity tends to increase the T*lag* value, a fact which may also be due to the larger size of the granulomas (Figures 3 and 4).

### Decrease in duplication time is advantageous to the bacilli

Evolution of the bacillary load with respect to the physiological values of T*dupli* in *M. tuberculosis* shows that reducing it to 16 hours results in a slight advantage as this doubles the number of bacilli (Figure S8).

Remarkably, a chimera made by reducing the T*dupli* to that of a hypothetical *M. tuberculosis* with a T*dupli* of 20 minutes (close to that of a more conventional bacteria such as *E. coli*; Figure S9) clearly shows that all these defensive mechanisms are totally useless when it comes to controlling such an organism. Indeed, even when only growing intracellularly, there is a major imbalance in the evolution of the infection which leads to a massive accumulation of extracellular bacilli.

### The degree of tolerance of the host influences the effect of the inoculum size

**Figure 7:**
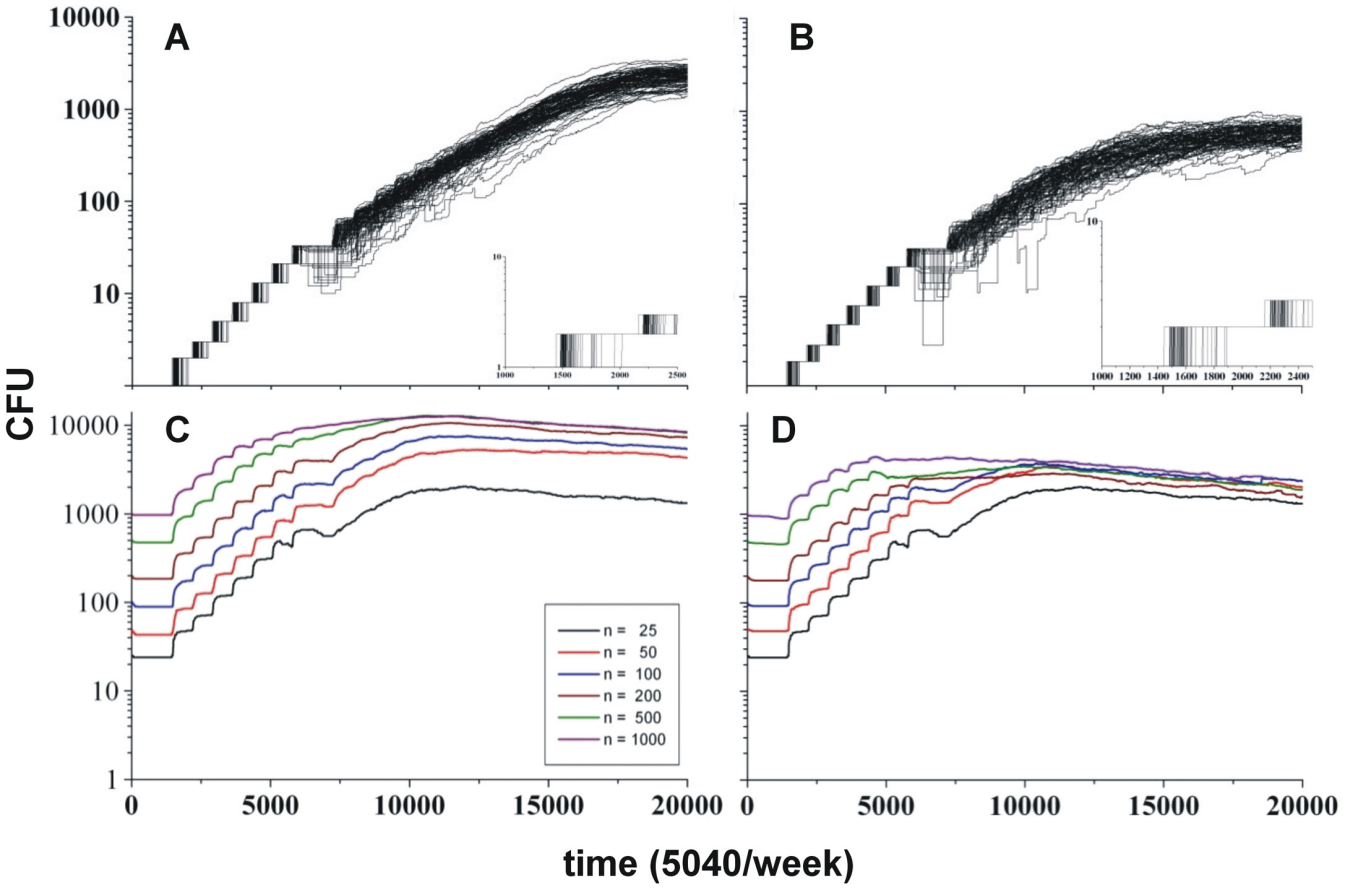
Importance of the initial bacillary load in the evolution of the infection. Importance of the initial bacillary load in the evolution of the infection. Pictures A and B show the evolution of 100 runs for HT and PT hosts with an immune response, respectively, inoculating with one bacillus in 100 naïve scenarios. Pictures C and D show the evolution of the total bacillary load when inoculated with different loads at one unique lattice.

Our study of the ability of a single bacillus to generate an infection in both HT and PT hosts, including the induction of adaptive immunity, showed that the bacilli in our system had a high ability to generate an infection and that this ability was higher for a HT than for a PT host (93% and 87% respectively; Figure 7 pictures A and B). This finding was also important to definitively validate the model and was used to extrapolate the infection to the whole lung, instead of a 4 mm^2^ lattice, by considering the inoculation of 50 independent 4 mm^2^ scenarios with one bacillus each, which is closer to the situation in a real-life aerosol infection. In the HT host, this resulted in an infection scenario with 1.1×10^3^ CFUs on average. If we multiply this by 10, considering a 3D parenchyma, and then by 50 scenarios, or pieces of lung, a total of 5.1×10^5^ CFUs are obtained, which fits the experimental “in vivo” data obtained with a low-dose aerosol in mice better[35], [53]. In the case of a PT host, the average was 2.6×10^5^ CFUs.

The influence of the inoculum size was also studied and the HT host again found to be more sensitive to this factor than the PT host, which showed a similar bacillary load after 20,000 iterations assuming onset of the adaptive immunity at the same time point (10,000 iterations, i.e. 2 weeks) (Figure 7, pictures C and D).

A re-infection process, which is a more similar scenario to a real-life situation whereby new infections usually arise in people living with the source of the infection (i.e. a patient with active TB who emits bacilli for two weeks before being diagnosed and then isolated and treated in a hospital, which means a hypothetical diagnostic delay of 15 days) was also considered. Thus, infecting every day for two weeks with 25 CFU (a total of 350 CFU) clearly resulted in a significant increase in the bacillary load (Figure S10A). When running this simulation, we again considered that the initial infection, or reinfection, took place in the same 4 mm^2^ lattice, which is thus a limiting factor in light of the importance of chemokine diffusion and granuloma formation for controlling the infection. When considering the onset of the infection for every bacillus in an independent lattice, and subsequently calculating the effect in a whole lung, we summed the evolution of independent growths to determine the bacillary count in the lung and ran this exercise by simulating 10 reinfection events, one every 24 hours for 10 days. This scenario obviously gave us a large bacillary load for the lung as a whole (5550 CFU) (Figure S10B).

### Induction of an effective humoral response significantly reduces the bacillary load

Figure S11 recreates a scenario in which the bacilli were attached to an antibody in a way that they could be destroyed with a specific degree of efficacy when phagocytosed. It must be noted, however, that this is the only defensive mechanism that can reduce the bacillary load at the very beginning of the infection and is thus a significant mechanism of protection that cannot yet be induced in LTBIs, but would clearly be useful if available.

## Discussion

A computational model that mimics the onset of *M. tuberculosis* infection on the basis of the low-dose aerosol model in C57BL/6 mice has been presented. This model explains why no lesions can be seen in the infected lung during the first weeks of the infection despite the constant increase in bacillary load. Furthermore, it also suggests that the chemokine concentration, and thus the ability of the infected macrophages to attract new cells, is crucial for controlling the infection and therefore that the ability to react to a certain concentration of these chemokines (known as “tolerance”) is also a paramount factor. In view of the results described above, it can be concluded that the host loses the battle against *M. tuberculosis* at the onset of the infection. Indeed, even when considering the presence of adaptive immunity or a PT host, it is impossible to avoid this window except in the presence of an effective humoral response.

This scenario explains perfectly why a person with a specific cellular immunity can be reinfected by *M. tuberculosis*, a fact which is supported by epidemiological and experimental data[57]. It also gives support to the theory that suggests that LTBI is considered to be a process of constant endogenous reinfection, and explains the induction of active TB as a process that depends on the site where the reinfection occurs, for example the upper lobes, favoring bacillary growth, liquefaction of the necrotic tissues and extracellular bacillary growth, which in turn lead to cavitation[51].

This model also confirms the role of the innate immune response in controlling the infection, including both the presence of NK and an apoptosis mechanism, and highlights the role of FM drainage as a powerful mechanism for innate control of the infection in the lung, although this mechanism requires large alveolar spaces, which are only found in large mammals[58].

Our study uses the same methodology as previous studies[9], although it differs notably as regards the conceptual framework of the infection. The main differences in this respect include the inclusion of the bacillary life cycle and not considering the extracellular bacillary growth as this is only possible when a liquefaction process takes place[17]. As far as the host response is concerned, macrophages are differentiated according to the phase of the bacilli, and this fact is also taken into account when defining the ability to present antigens and activate the macrophage. Overall the relationship between the macrophages and the bacilli is better defined and avoids concepts such as “chronically infected” macrophages, which are difficult to fit into traditional “in vitro” and “in vivo” experimental models. We also consider the presence of neutrophils, although with only a “neutral” role in terms of macrophage activation as their role in this process is still controversial[21], [23]. However, we include neutrophils as an important factor in terms of fixing the bacilli at the site where the infected macrophage necroses or apoptoses, space occupation, and also as an inducer of FM once apoptosed. This is a key factor in our model as an innate defense mechanism that involves draining non-replicating bacilli towards the alveolar spaces in large mammals and also as a source of the bacilli which induce the constant endogenous reinfection process that forms the basis of the “dynamic hypothesis” proposed to explain LTBI in large mammals[51]. These cells mainly remain in the FMs as a future source of granulomas and the progressive infiltration seen in the mice model[59]. We have also included the presence of NK as agents that can induce an innate protection. This is an important factor as it has been demonstrated experimentally that some degree of bacillary destruction occurs during this phase[20].

Our model fits well with the best known animal model, which involves disease induction after a low-dose aerosol in mice. Published data clearly demonstrate that granulomas are rarely, if ever, seen before week 3[35], [60]. Furthermore, unpublished data obtained by our group (Vilaplana et al., manuscript in preparation) after infecting C57BL/6 mice with a low-dose aerosol (around 50 CFU) show that a very small number of microscopic granulomas can be seen before week 3 post-infection despite the fact that the onset of the immune response and the control of the bacillary load in the lungs effectively takes place on day 15 post-infection. The size of these granulomas (less than 100 µm) places them at the limit between a cellular accumulation and a granuloma and raises questions regarding the granuloma concept itself. Some extracellular bacilli were also observed after an exhaustive and careful examination of the whole lungs of these mice, which were harvested on a daily basis from day 10 to day 21 post-infection. These findings are of great interest as they suggest that the granulomatous process is not specifically related to the adaptative immune response but can also take place through the innate response, as already predicted in a model of *M. marinum* infection in zebrafish[61]. Furthermore, another phenomenon in this model, namely the presence of a diffuse cellularity, tended to disappear with the onset of adaptive immunity, in other words when the granulomas increased in size.

This simulation reproduces the initial evolution of the infection, including onset of the infection and of the adaptive immune response. Previous studies did not address this issue and focused in making predictions regarding the chronic phase (around 500 days post-infection). This can be considered to be a major drawback as this period is vital to understanding a key characteristic of *M. tuberculosis* infection, namely that only the cellular immune response is able to give protection in a process which requires the DC to give a positive feedback and induce the proliferation of specific T cells at the lymph nodes to start the activation of the infected macrophages[29], [30]. This drawback has been discussed previously, and means that vaccination to induce an adaptive cellular immune response can only reduce the CFU counts rather than prevent the infection itself[32], thereby indicating that a hypothetical effective humoral response is likely to be the only means of preventing infection. In this regard, many authors have already demonstrated the ability of antibodies to give such protection when administrated in a passive way[62], [63], although much work remains to be done in order to obtain such a vaccine and an effective immune memory based on these hypothetical protective antibodies.

Our model also highlights the importance of the local milieu, in other words the chemokine factor that is needed to attract the specific lymphocytes to activate the infected macrophages. We have detected this problem in a very small ideal scenario (4 mm^2^) as the surface of an adult lung consists of up to 320,000 such scenarios (160 m^2^, around three-quarters of a tennis court)[64]. It should be stressed that we have simplified the chemokine mechanism to a very basic and ideal one and have not taken into account all the amplification mechanisms that make this attraction possible. However, this model at least reflects what happens in mice in terms of CFU and granuloma formation and chemokine expression[35], [65]. Furthermore, it also mimics what happens in what we consider to be a PT model, namely mini-pigs, where the bacillary load tends to decrease rather than increase with time after inoculation of 10^3^ bacilli directly into the lung parenchyma[56]. It is well known that alveolar macrophages are "tolerant" in that they tend to cause immunosupression or follow an alternative activation pathway, although this must be considered in a holistic manner as the alveolar space is always full of inhaled bacteria, virus and toxic particles. However, the behavior of the alveolar macrophages whereby they maintain the local homeostasis would appear to be logical as a more aggressive behavior would always result in a damaged lung.

Our model can also be used to study the whole lung instead of just a single lattice by considering one “naïve” lattice for each infective bacilli. The results suggest a significant role for the reinfection process, thereby supporting the “dynamic hypothesis” and enabling us to better understand the reality of scenarios with a high prevalence of active TB patients where there is a very high probability of constant exogenous reinfection. It is interesting to note that this is possible even in the presence of constantly available circulating specific T cells, which is the scenario found in LTBI patients, as can be seen with the recently developed range of diagnostic tools based on the detection of these cells in peripheral blood using the TIGRAS method.

At this point is important to highlight the limitations of our model. In the majority of cases we have only been able to make predictions up to four weeks post-infection as other factors, such as re-growth of the non-replicating bacilli inside the FM, as observed in the mouse model[34], [35], or the key role played by the fibrosis and encapsulation of the granulomas observed in the mini-pig model[56], as also noted by Canetti[66], that complicate progression of the disease can come into play at this point. The first example would imply a constant increase in bacillary load which would therefore require a bigger lattice, whereas the second case would require the inclusion of another important factor, namely fibroblasts, and also physical contact with the intralobular septae. Such a change would probably also require the inclusion of another type of fibroblast that is able to synthesize type I collagen, together with the mineralization process that results in the multistress phenomenon for the extracellular bacilli embedded in the necrotic tissue[56]. Despite the fact that all these factors are complicated and still not fully understood, they probably lead to a better control of the bacillary load.

Another limitation concerns the anatomic scenario. We have considered a “solid” organ, which is clearly not the case for the lungs with their alveolar spaces, alveolar wall, endothelial and other epithelial cells, including fibroblasts, which represent 30–40% of the total number of cells[67]. Such a scenario would, however, be very difficult to simulate in terms of space occupation and would also require a great deal of imagination to recreate how the alveolar walls are destroyed and how the fibrin forms a physical platform to occupy the empty alveolar spaces. This would lead to a highly complex model, although it may well be possible to develop such a model in the future once new experimental data become available.

This model also fails to take into account the role of the innate ability or the unspecifically activated macrophages to destroy new incoming bacilli from the very beginning, a factor that should be included to reduce the assumed 87% likelihood of a single bacillus initiating an infection. Indeed, it has been estimated that this value in humans is only around 30% [2]. This is also, however, a complex scenario which includes a huge number of events that can hamper the ability of the bacilli to establish the infection, especially the role of the different elements contained in the surfactant to impair the virulence of the bacilli before they come into contact with the macrophage, or the status of the macrophage itself when facing the bacilli. We have assumed a scenario in which all the alveolar macrophages (resting macrophages) available are totally naïve from the outset. This is probably not the case, however, as these cells are constantly challenged with pathogens and toxic elements that are constantly introduced into the alveolar space upon respiration and which are also likely to induce some kind of activation.

Furthermore, when recreating the “human model” as a PT host we have only assigned the higher reactivity to the presence of chemokines, which, of course, may not actually be the case. Our model therefore places significant emphasis on the NK and their ability to activate infected macrophages despite the likelihood that numerous other factors also play a part. Indeed, the properties of the macrophages themselves may differ from those found in mice and the activation percentages may also differ between the two species. We have only changed one factor despite the fact that this is highly likely to be a multifactorial process. However, we wanted to highlight how important this factor could be for controlling the infection and the fact that such control is clearly higher in humans than in mice as all infected mice die as a result of absolute infiltration of the lung, whereas, at most, only 40% of humans do so if active TB is triggered, which only occurs in 10% of cases. The differences found between the results obtained in the in silico model regarding the chemokine levels and their influence on infection control could be the explanation to explain this process.

Our representation of a generic chemokine and the “activation process” is necessarily an oversimplification of these highly complex processes as the addition of such complexity would make the model even less reliable. For this reason we have mainly focused on a relatively primitive process and tried to fit it to easily measureable parameters, such as granuloma size and bacillary counts in the murine experimental model.

On the basis of previous studies, we have attempted to develop a model to explain our current understanding of this disease process but which will nevertheless have to be modified in the future to take into account new experimental data. However, despite the current limitations of this model, we strongly believe that some basic elements, such as the impossibility of curtailing the initial growth of the bacilli, might help to structure current data regarding the infection process and to develop new tools and strategies to better understand and combat it.

Finally, our model also does not take into account the reinfection process resulting from the drainage of bacilli from infected lymph nodes, via the lymph ducts, to the right atrium and the pulmonary artery and from there back to the lung.

In conclusion, the data presented herein are an attempt to combine, in a limited scenario, the singularities of the cellularity and the infection process in a cellular automata model to simulate the infection process in mice and extrapolate it to a “human-like” model whilst also taking into account the life-cycle of the bacilli and the concept of “host tolerance”. Overall, this model helps to understand the mechanisms of *M. tuberculosis* persistence in the host on the basis of its slow metabolism, its ability to survive stressful conditions, and the cellular nature of the adaptive immunity triggered, which leaves a number of “windows” open to the bacilli that complement its natural ability to persist.

## Materials and Methods

### Scenario

**Figure 8:**
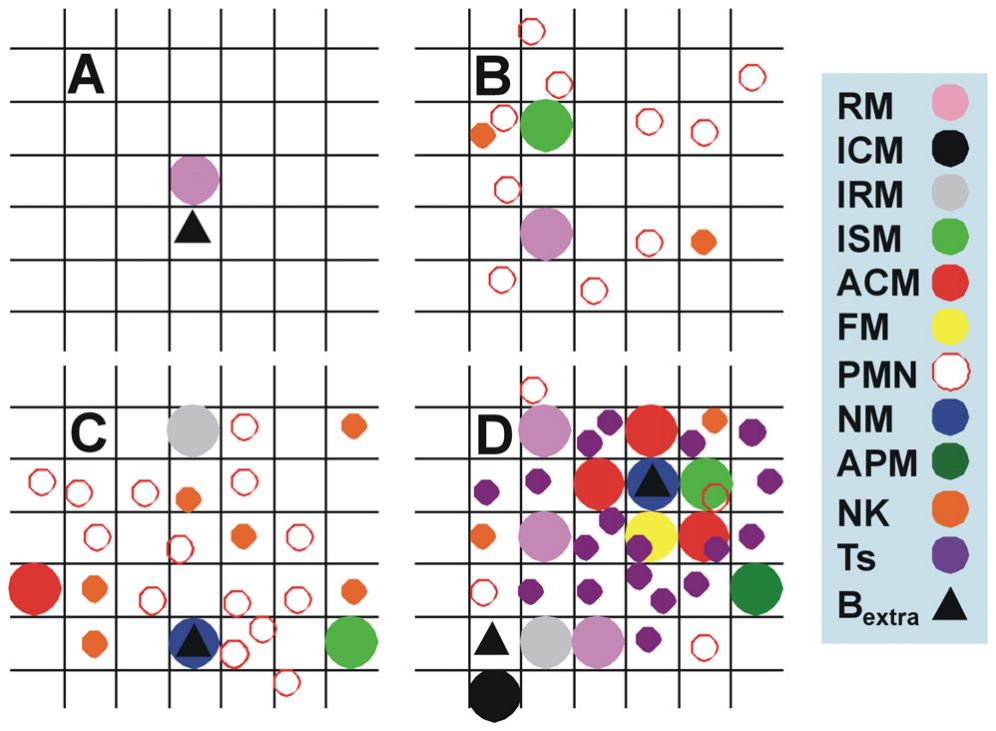
Physical scenario of the cellular automata simulation system. Physical scenario of the cellular automata simulation system. A two-dimensional 100×100 lattice of micro-compartments has been built. As the diameter of an alveolar macrophage is around 20 µm, each of the micro-compartments in the lattice represents a square with dimensions 20×20 µm. A maximum of three live or dead cells can fit in each square, although only one living macrophage can be in a square at any one time (i.e. two living macrophages cannot be in the same square). The following pictures reproduce different phases in the evolution of the model. Picture **A** shows the very beginning of the infection, namely extracellular bacilli and a resting macrophage; **B** shows an infected macrophage that has already attracted cells around it; **C** shows a necrosed macrophage caused by the intracellular growth of the bacilli; **D** shows the entrance of immune response once the granuloma has been formed and the appearance of foamy macrophages.

A two-dimensional 100×100 lattice of micro-compartments was constructed to mimic a granuloma. As the diameter of an alveolar macrophage is around 20 µm, each of these micro-compartments represents a square with dimensions 20×20 µm. A maximum of three live or dead cells can fit into each square, although only one living macrophage can be present at any one time (i.e. two living macrophages cannot be present in the same square; Figure 8). This scenario represents a 4 mm^2^ surface which is able to contain a primary lesion in the experimental murine or guinea pig experimental models[54], [68] and at least the granulomas generated after the immune acquisition in bovine[69], primate[70] and mini-pig models[56].

### Cellular propagation speed and lifetime

Time is one of the key dimensional parameters in any computational simulation. In this model, time is introduced by considering finite time-steps during which the rules are applied to all the cells in the grid to generate a totally new scenario. We decided to run a iteration (time-step) every two minutes of real time, which, according to Miller et al[71], is the approximate time required for a lymphocyte to move a distance of 20 µm. The rate of macrophage movement was determined on the basis of previous experimental data[9] and was considered to be an average of 1 µm/minute (Table 1). The direction in which the cells moved was determined by the chemokine gradient at every time-step or by random walk in absence of chemokines. The lifetimes for macrophages, neutrophils and lymphocytes were taken to be 3 months, 3 days and 3 hours respectively [9], [24] (Table 1).

**Table 1:** Lifetime and speed of the different cellular entities.

Cell	Lifetime		Speed	
	Time	Iterations	µm/minute	Iterations/compartment*
RM	100 days	72,000	1	10
IM				
ICM				
IRM				
ISM				
ACM				
NM	N.A.			
APM				
PMN	3 hours	90	10	1
NK	3 days	2160	10	1
Ts				

Macrophages =  resting (RM); infected (MI); infected-consolidated (ICM); infected-at risk (IRM); infected-sentenced (ISM); activated (ACM); necrosed (NM); apoptosed (APM).

Neutrophils (PMN); Natural killers (NK).

Specific T lymphocytes (Ts).

N.A. =  not applicable.

Iterations =  Time-steps.

*number of iterations required to move the distance of one micro-compartment of the lattice.

### System entities and the rules that govern them

**Figure 9:**
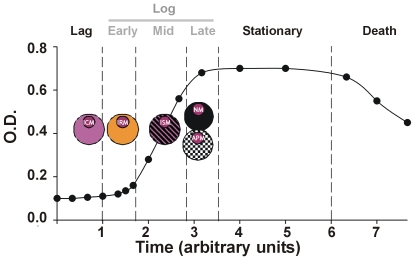
Characterization of the macrophages. Characterization of the macrophages according to the most advanced phase of the life cycle of the bacilli inside them. The bacillary life cycle evolution, as monitored using the optical density in a static liquid culture, is represented in the background. (Modified from Buchanan 1918[40]).

Four groups of entities were considered: a generic chemokine, bacilli, macrophages and other cells (Figures 8, 9, S12). Initially, a total of around 100 cells/mm^2^ of resting macrophages (***RM***) and 1 cell/mm^2^ of natural killers (***NK***) was considered to be present[9], [72]. Chemokines are treated as a continuous variable that peaks at the lattice where they are generated and spread in a gradient across the neighboring lattices (Figure S10). Macrophages, other cells and bacilli are represented as discrete agents.

#### Chemokine and granuloma formation

We decided to consider the macrophages as the only source of a hypothetical global chemokine and that the concentration of chemokines produced depended on the evolutive state of the macrophage (Table 2). Thus, we considered that macrophages harbouring metabolically active bacilli secreted more chemokines, and that necrotic macrophages induced a single strong peak, according to their strong inflammatory role[73], [74].

**Table 2:** Ability of the different cellular entities to generate chemokines (in arbitrary units).

Cell	Peak production			Continuous Production
	Transition to	Post bacterial		
		Phagocytosis	Killing	
ICM	100	100	N.A.	100
IRM	500	500		
ISM	500	500		
ACM	0	0	100	
NM	10,000	N.A.	N.A.	N.A.

Macrophages =  infected-consolidated (ICM); infected-at risk (IRM); infected-sentenced (ISM); activated (ACM); necrosed (NM).

N.A. =  not applicable.

We also assumed that all chemokines secreted diffuse throughout the network as assumed by others [9], with a decay term, δ, represented by the continuous equation:
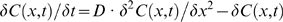
where D is the diffusion coefficient of chemokines and δ is a decay coefficient related to the half-life of the chemokine IL-8. The numerical values were constant: δ = 0.00577 min^−1^ and δC = 0.4. To determine the exact value of the chemokine concentration in any compartment *(i,j)* of the network, we discretized this equation using a conventional finite differences scheme, with *Dt = 2 min* and *Dx = 2.10^−5^ m*. We considered that the chemokines can diffuse to the four adjacent compartments *(i+1,j), (i-1,j), (i,j-1)* and *(i,j+1)* and imposed periodic boundary conditions on the network.

The chemokine gradient attracts the cells towards the highest concentration, modifying their random movement accordingly, and induces the generation of a new cell in every square where the chemokine concentration is above a certain threshold (**CT**).

In our model, we adapted the concept of host tolerance[55], namely the ability to react and generate a new cell according to a certain **CT**, which decreases as the host becomes less tolerant. Determination of chemokine values and threshold was performed on the basis of the size of the lesions found in experimental models. The guideline considered when adjusting such parameters was to obtain lesions with a diameter of at least 0.4 mm at three weeks post-infection, as has been described in mice, guinea pigs, cows, mini-pigs and monkeys[54], [56], [68], [69], [70].

At every time-step, the system checks the chemokine concentration in each micro-compartment. As indicated above, cells then move towards the highest concentration, and if this is above a determined **CT** a new cell will appear in that compartment (if it is not already occupied). This is coherent with the proposal that there is a dense capillary net in the alveolar parenchyma. The type of cell that appears varies according to the status of the host's immunity, as specified in Table 3, taking into account data obtained from experimental models that give a certain proportion at discrete time-points in the bronchoalveolar lavage or the whole lung[72]. The host is considered to have adaptive immunity two weeks post-infection, as is usually observed in the mice model[9], [75].

**Table 3:** Probability (in %) of cellular appearance in a compartment once the chemokine concentration is above the cellular threshold.

Cells	Immunity	
	Innate	Specific
RM	90	99.74
PMN	8	0.05
NK	2	0.01
Ts	0	0.2

RM =  resting macrophages; PMN =  neutrophils; NK =  Natural killers; Ts =  specific T lymphocytes.

#### Bacilli

According to observations in different experimental models, we have assumed that only intracellular bacilli residing in a non-activated macrophage are able to grow[17], [45] [76], [77] and H37Rv considered as the infective *M. tuberculosis* strain. The doubling time is between 16 and 24 hours[78], and this is reached at the mid-*log* phase (Figure 9). We determined that once the growing bacilli reach a certain concentration (around 32 bacilli) [Lee, personal communication], the macrophage becomes necrotic. We also determined that this growth could be stopped by apoptosis of the macrophage (which occurred with a certain probability, also obtained from experimental data[74], [79]), a mechanism that we considered could be triggered once the number of bacilli had reached 16. The fate of the bacilli is different in both cases. After apoptosis, there is a brief length of time corresponding to the late-*log* phase (about 24 hours) when these bacilli transform from the *log* to the stationary phase (Figures 9 and 11) and can be killed by any macrophage once phagocytosed irrespective of their activation status[79]. In the event of macrophage necrosis, we considered a different scenario whereby only already activated macrophages can kill these bacilli in the late-*log* phase. After this period, the bacilli become ***Bextra*** and therefore highly resistant to further stressful conditions[20], [46], [80], [81].

**Figure 10:**
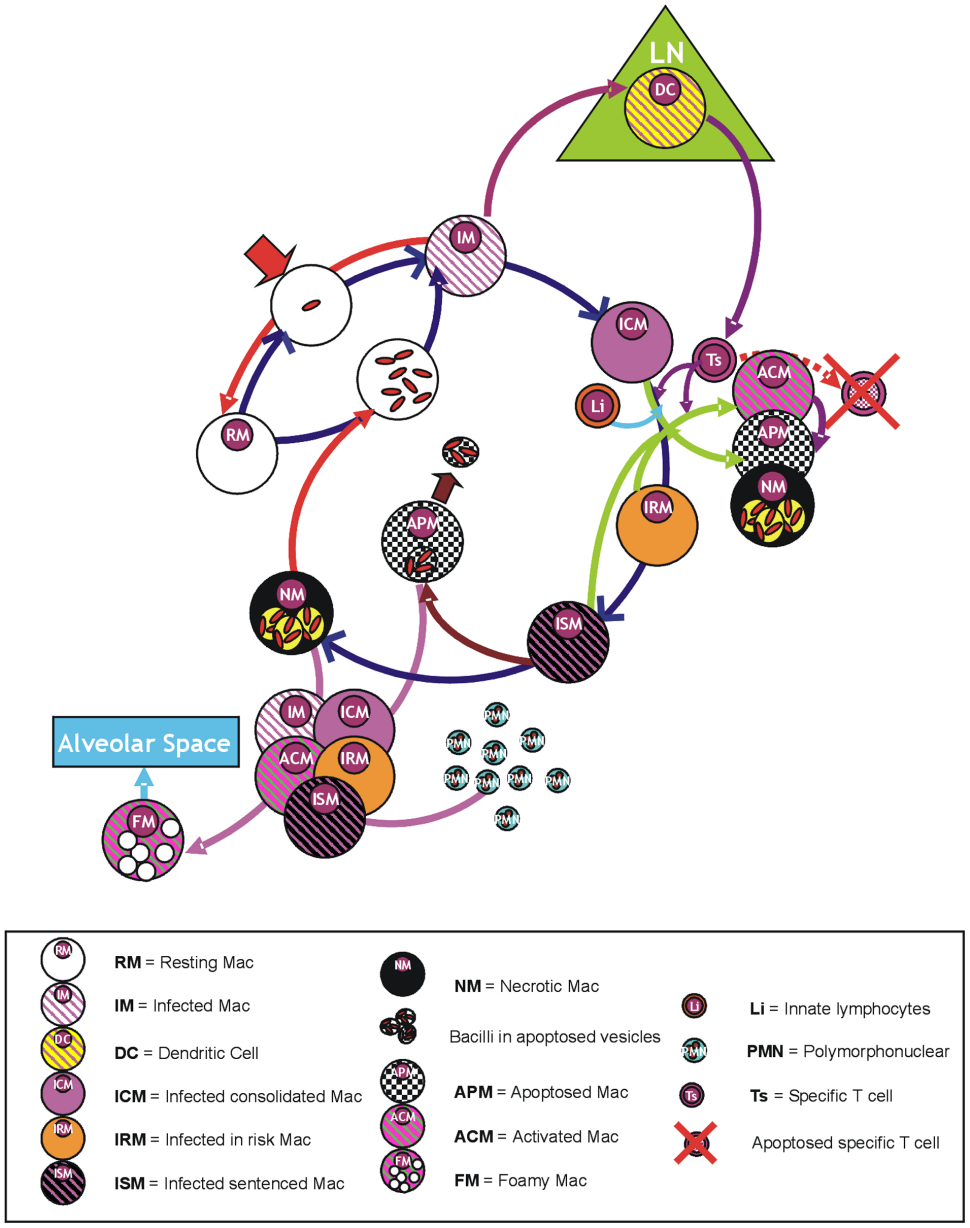
*Mycobacterium tuberculosis* infection cycle. Representation of the *M. tuberculosis* infection cycle according to the macrophage phase status (infection, activation, necrosis, apoptosis and foamy macrophage production). Differentiation to dendritic cells is also considered at the very first phase of the evolution.

**Figure 11:**
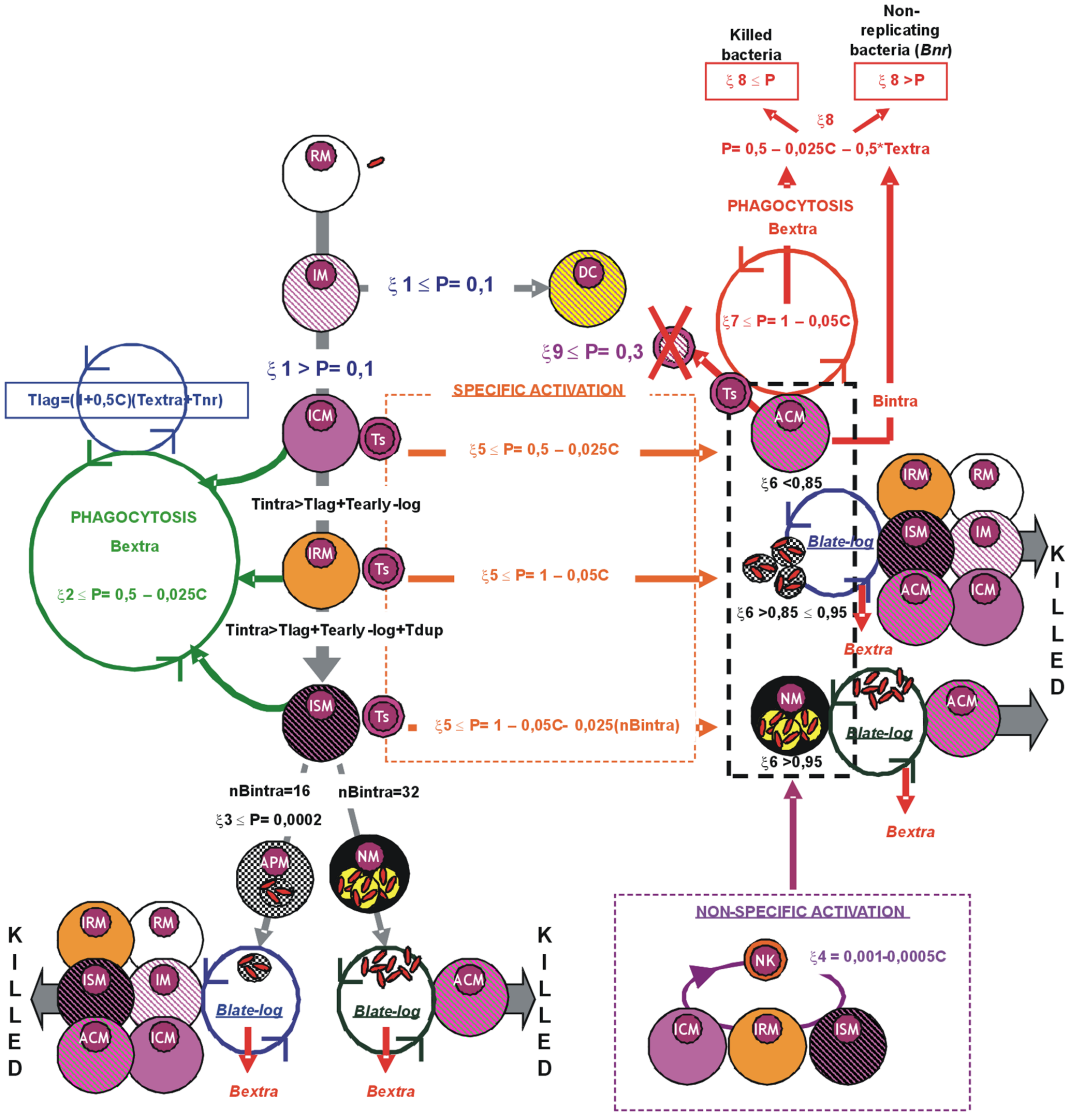
Transformation of the macrophage status. Transformation of the macrophage status according to given probabilities, taking into account the number of cells (C) and bacilli (nB*intra*) phagocytosed; the time that a bacilli has been in the extracellular milieu (Textra) or inside the macrophage (T*intra*); and the bacillary life-cycle periods (i.e. T*lag*, T*early-log* and T*late-log*) together with the bacillary duplication time (T*dupli*). The presence of intra- or extracellular bacilli (B*intra* and B*extra* respectively), as well as when a bacteria becomes non-replicating for a long time (B*nr*) and the time that it maintains this status (T*nr*), are also indicated.

We quantified the time that the bacilli remain in the extracellular milieu and considered that this should determine the length of their *lag* phase (*Tlag*)[18], [47] (once phagocytosed again by a non-activated macrophage) and thus the time to start their metabolism (Figures 10 and 11). This is because the extracellular milieu is stressful for the bacteria as become involved with anoxia, toxic fatty acids, and low pH [18]. These stresses has been experimentally related to the increase on the lag phase in other bacterial intracellular pathogens [48]. After this time, the bacilli then start the early-*log* phase proportionally to T*dupli* [49], i.e. 24 hours, when the bacilli are still not duplicating. Once at mid-*log* phase and the duplication time has passed, there will be two children bacilli.

When a ***Bextra*** was phagocytosed by an already activated macrophage, it was considered to remain in a non-replicating state (***Bnr***) for a long period or to be killed. The only opportunity for *Bnr* to become extracellular again was when the macrophage died due to being old. In this case, once these bacilli were phagocytosed again by a non-activated macrophage, their *Tlag* was determined according to the time spent as ***Bnr*** (***Tnr***) plus the time they had spent as ***Bextra*** (***Textra***; Figures 10 and 11).

We also considered that the ability to phagocytose and kill ***Bextra*** was dependent on the current cellular charge (***C***; Figure 11) a value determined by the number of necrotised cells phagocytosed (i.e. one *c* for each necrotised neutrophil; three *c* for each necrotised macrophage), as it is the capacity to kill the bacilli once phagocytosed by already activated macrophages. Only one ***c*** can be phagocytosed at every time-step when the macrophage touches the cell where the necrotised cell can be found.

#### Evolution of the macrophages

As stated above, the established rule was that both live and dead cells occupied the space until they moved or were phagocytosed, respectively. Each compartment could therefore contain three cells, either alive or dead. The only exclusion criterion was that a compartment could only hold one living macrophage (i.e. two living macrophages could not fit in a single compartment). Phagocytosis of dead cells took place once a cell entered the square where the dead cells were located or by being in one of the four squares that had a side in common.

Macrophages were classified according to the life-cycle status of the interior bacillus with the most active metabolism. There was no constitutive entrance of macrophages. Resting macrophages (***RM***) could be infected (***MI***), with 10% becoming dendritic cells (***DC***) and migrating towards the lymphocytic vessels (Figures 3 and 5)[26], [27], [28]. The remainder became infected-consolidated (***ICM***), and remained in this state until one of the intracellular bacilli had been inside the macrophage for a period of time (***Tintra***) longer than the established ***Tlag*** (i.e. *Tintra*>*Tlag*). At this point the early-*log* (*Tleg*) stage appeared to prepare the metabolic machinery for growth. The macrophages were now “at risk” (***IRM***) of harboring intracellular growth, when secreted antigens can start to be presented and there is thus a higher chance of becoming activated (Figure 11). After this *Tlag* (considered to be equivalent to the bacillary doubling time, i.e. 24 hours), all the intracellular bacilli started to grow and took another 24 hours to effectively double in number, at which point the macrophage became sentenced (***ISM***) as it had had the maximum chance to die (Figure 11).

Once a macrophage was necrosed (***NM***) or apoptosed (***APM***), the bacilli remained in the same micro-compartment. However, only one bacillus could be phagocytosed during each time-step that a macrophage “touched” or entered the compartment.

Once an NK cells or specific lymphocyte (***Ts***; we did not distinguish between CD4 or CD8 cells) touched a micro-compartment containing an infected macrophage, this macrophage could be activated (***ACM***), apoptosed (***APM***) or necrosed (***NM***). This probability depended on the nature of the activator cell (**NK** or **Ts**) and the evolutive state of the macrophage and the current cellular charge (C) (Figure 11).

Although induction of adaptive immunity is thought to be triggered once a specific concentration of ***DCs*** reach the regional lymph node[29], [30], we simplified this to be induced two weeks (i.e. 10.000 iterations) post-infection, as occurs in the experimental murine model[32], [75].

If a specific lymphocyte (***Ts***) touched an already activated macrophage, this had a probability (*P* = 0.3) of being apoptosed, according to the data of Holt[26].

Dead cells occupied space (as seen above) until phagocytosed by a macrophage. Each dead neutrophil represented one ***C***, and every macrophage filled the equivalent of 3***C***. Once a macrophage had phagocytosed 21 or more C's it became an **FM** and could not phagocyte any more cells. In the case of the PT host, which is thought to include all large mammals, when the four neighboring compartments limiting the four sides of a compartment filled by a FM were empty, the FM was considered be out of the lattice, simulating that it had reached an open alveolar space, and was drained towards the upper bronchi.

We have not modified the speed capacity of the macrophages according to their status as others made before [9], as there is no evidence that activation or infection decreases the motility of the alveolar macrophages, on the contrary, apparently it could be increased[82].

## Supporting Information

Figure S1 Evolution of the drainage of Foamy Macrophages (FM) in poorly tolerant (PT) hosts, according their immunity status
(5.93 MB TIF)

Figure S2 Photomicrographs of infected lungs in experimental models induced in mice (A to C) and mini-pigs (D to F) at weeks 3 and 5 respectively. Cuts were stained with haematoxylin-eosin (A to E) or visualized with a stereoscopic microscope, in the case of mini-pigs (F).
(5.40 MB TIF)

Figure S3 Macrophage infiltration and chemokine concentration in the space at week 4 post-infection in the case of a poorly tolerant host with an immune response. A: macrophage infiltration; B: chemokine production; C: combined image.
(3.66 MB TIF)

Figure S4 Evolution of the total amount of chemokines with time in all the cases studied.
(5.93 MB TIF)

Figure S5 Influence of the apoptosis ratio on the bacillary concentration. The usual probability is compared with a 10-fold increase in the possibility of apoptosis, including the evolution of the numbers of necrotic and apoptotic macrophages in both cases (small squares).
(5.86 MB TIF)

Figure S6 Evolution of dendritic cell (DC) formation in all the cases studied.
(6.57 MB TIF)

Figure S7 Distribution of Tlag for the four cases studied. Data are presented as boxes showing the 25th and 75th percentiles, and the 10th and 90th percentiles with error bars. The median is shown as a horizontal line inside the boxes. Differences between groups were determined using an all pairwise multiple comparison procedure (Dunn's Method), and are marked with * when significant.
(8.72 MB TIF)

Figure S8 The influence of Tdupli and extracellular growth on the evolution of the total bacillary load, showing the influence of different Tdupli values.
(5.96 MB TIF)

Figure S9 Recreation of a bacillary "chimera" with Tdupli = 20 minutes.
(5.88 MB TIF)

Figure S10 Role of reinfection in the evolution of the infection. Role of reinfection in the evolution of the infection. Picture A shows the standard inoculation in a PT host with immunity compared with the same host when constantly reinfected with 25 CFUs until t = 10,000 in the same lattice. Picture B shows the evolution of a whole lung of a person reinfected just 10 times with one bacillus in 10 different naïve lattices. The bacillary load is 5550.
(10.17 MB TIF)

Figure S11 Presence of an hypothetical efficacious humoral response. Induction of a humoral response allowing the bacillus to be killed by any macrophage that phagocytes the opsonized bacillus, considering different percentages of activity (shown in the legend insert).
(6.16 MB TIF)

Figure S12 Dissemination of a single chemokine peak through space at different time points.
(5.82 MB TIF)

Video S1 Simulation in a Highly tolerant host. Left window shows the evolution of the chemokine concentration; right window shows the evolution of the granuloma formation were entities are: RM (resting macrophage) in pink; ICM, IRM, ISM (infected consolidated, risk and sentenced macrophages) in green; NM (necrotic macrophages) in black; FM (foamy macrophages) in yellow; and ACM (activated macrophages) in red. Timing appears at the bottom right in steps.
(3.03 MB AVI)

Video S2 Simulation in a Highly tolerant host. Left window shows the evolution of the chemokine concentration; right window shows the evolution of the granuloma formation were entities are: RM (resting macrophage) in pink; ICM, IRM, ISM (infected consolidated, risk and sentenced macrophages) in green; NM (necrotic macrophages) in black; FM (foamy macrophages) in yellow; and ACM (activated macrophages) in red. Timing appears at the bottom right in steps.
(3.11 MB AVI)

Video S3 Simulation in a Poorly tolerant host showing the evolution of the granuloma formation were entities are: RM (resting macrophage) in blue; ICM, IRM, ISM (infected consolidated, risk and sentenced macrophages) in green; NM (necrotic macrophages) in black; FM (foamy macrophages) in yellow; ACM (activated macrophages) in red; and Ts (specific lymphocytes) in cerulean blue. Timing appears at the bottom right in steps.
(3.74 MB AVI)

Video S4 Simulation in a Poorly tolerant host showing the evolution of the granuloma formation were entities are: RM. ICM, IRM, ISM, ACM (resting, infected consolidated, risk and sentenced and activated macrophages) in green; NM (necrotic macrophages) in black; FM (foamy macrophages) in yellow; and Bextra (extracellular bacilli) in red. Timing appears at the bottom right in steps.
(3.39 MB AVI)

## References

[1] Cardona P, Ruiz-Manzano J (2004). On the nature of Mycobacterium tuberculosis-latent bacilli.. Eur Respir J.

[2] Parrish NM, Dick JD, Bishai WR (1998). Mechanisms of latency in Mycobacterium tuberculosis.. Trends Microbiol.

[3] WHO (2009). Global tuberculosis control: epidemiology, strategy, financing: WHO report 2009..

[4] North RJ, Jung YJ (2004). Immunity to tuberculosis.. Annu Rev Immunol.

[5] Andersen P (1997). Host responses and antigens involved in protective immunity to Mycobacterium tuberculosis.. Scand J Immunol.

[6] Peyron P, Vaubourgeix J, Poquet Y, Levillain F, Botanch C (2008). Foamy macrophages from tuberculous patients' granulomas constitute a nutrient-rich reservoir for M. tuberculosis persistence.. PLoS Pathog.

[7] Egen JG, Rothfuchs AG, Feng CG, Winter N, Sher A (2008). Macrophage and T cell dynamics during the development and disintegration of mycobacterial granulomas.. Immunity.

[8] Otto SP, Day T (2007). A biologist's guide to mathematical modeling in ecology and evolution..

[9] Segovia-Juarez J, Ganguli S, Kirschner D (2004). Identifying control mechanisms of granuloma formation during M. tuberculosis infection using an agent-based model.. J Theor Biol.

[10] Vergne I, Chua J, Lee H, Lucas M, Belisle J (2005). Mechanism of phagolysosome biogenesis block by viable Mycobacterium tuberculosis.. Proc Natl Acad Sci U S A.

[11] Russell DG (2007). Who puts the tubercle in tuberculosis?. Nat Rev Microbiol.

[12] Roberts E, Deretic V (2008). The Mycobacterium tuberculosis phagosome.. Methods Mol Biol.

[13] Park JS, Tamayo MH, Gonzalez-Juarrero M, Orme IM, Ordway DJ (2006). Virulent clinical isolates of Mycobacterium tuberculosis grow rapidly and induce cellular necrosis but minimal apoptosis in murine macrophages.. J Leukoc Biol.

[14] Hemsworth GR, Kochan I (1978). Secretion of antimycobacterial fatty acids by normal and activated macrophages.. Infect Immun.

[15] Leemans JC, Thepen T, Weijer S, Florquin S, van Rooijen N (2005). Macrophages play a dual role during pulmonary tuberculosis in mice.. J Infect Dis.

[16] Martinez D, Vermeulen M, von Euw E, Sabatte J, Maggini J (2007). Extracellular acidosis triggers the maturation of human dendritic cells and the production of IL-12.. J Immunol.

[17] Grosset J (2003). Mycobacterium tuberculosis in the extracellular compartment: an underestimated adversary.. Antimicrob Agents Chemother.

[18] Converse P, Dannenberg AJ, Estep J, Sugisaki K, Abe Y (1996). Cavitary tuberculosis produced in rabbits by aerosolized virulent tubercle bacilli.. Infect Immun.

[19] Sada-Ovalle I, Chiba A, Gonzales A, Brenner MB, Behar SM (2008). Innate invariant NKT cells recognize Mycobacterium tuberculosis-infected macrophages, produce interferon-gamma, and kill intracellular bacteria.. PLoS Pathog.

[20] Gill WP, Harik NS, Whiddon MR, Liao RP, Mittler JE (2009). A replication clock for Mycobacterium tuberculosis.. Nat Med.

[21] Zhang X, Majlessi L, Deriaud E, Leclerc C, Lo-Man R (2009). Coactivation of Syk kinase and MyD88 adaptor protein pathways by bacteria promotes regulatory properties of neutrophils.. Immunity.

[22] Tan BH, Meinken C, Bastian M, Bruns H, Legaspi A (2006). Macrophages acquire neutrophil granules for antimicrobial activity against intracellular pathogens.. J Immunol.

[23] Persson YA, Blomgran-Julinder R, Rahman S, Zheng L, Stendahl O (2008). Mycobacterium tuberculosis-induced apoptotic neutrophils trigger a pro-inflammatory response in macrophages through release of heat shock protein 72, acting in synergy with the bacteria.. Microbes Infect.

[24] Brinkmann V, Zychlinsky A (2007). Beneficial suicide: why neutrophils die to make NETs.. Nat Rev Microbiol.

[25] Lenzi H, Romanha WS, Santos R, Rosas A, Mota E (2006). Four whole-istic aspects of schistosome granuloma biology: fractal arrangement, internal regulation, autopoietic component and closure.. Mem Inst Oswaldo Cruz.

[26] Holt P (2000). Antigen presentation in the lung.. Am J Respir Crit Care Med.

[27] Holt P, Stumbles P (2000). Characterization of dendritic cell populations in the respiratory tract.. J Aerosol Med.

[28] Serbina N, Jia T, Hohl T, Pamer E (2008). Monocyte-mediated defense against microbial pathogens.. Annu Rev Immunol.

[29] Chackerian AA, Alt JM, Perera TV, Dascher CC, Behar SM (2002). Dissemination of Mycobacterium tuberculosis is influenced by host factors and precedes the initiation of T-cell immunity.. Infect Immun.

[30] Wolf AJ, Desvignes L, Linas B, Banaiee N, Tamura T (2008). Initiation of the adaptive immune response to Mycobacterium tuberculosis depends on antigen production in the local lymph node, not the lungs.. J Exp Med.

[31] Flynn JL, Chan J (2001). Immunology of tuberculosis.. Annu Rev Immunol.

[32] Jung Y, Ryan L, LaCourse R, North R (2005). Properties and protective value of the secondary versus primary T helper type 1 response to airborne Mycobacterium tuberculosis infection in mice.. J Exp Med.

[33] D'Avila H, Roque NR, Cardoso RM, Castro-Faria-Neto HC, Melo RC (2008). Neutrophils recruited to the site of Mycobacterium bovis BCG infection undergo apoptosis and modulate lipid body biogenesis and prostaglandin E production by macrophages.. Cell Microbiol.

[34] Cardona PJ, Llatjos R, Gordillo S, Diaz J, Ojanguren I (2000). Evolution of granulomas in lungs of mice infected aerogenically with Mycobacterium tuberculosis.. Scand J Immunol.

[35] Cardona PJ, Gordillo S, Diaz J, Tapia G, Amat I (2003). Widespread bronchogenic dissemination makes DBA/2 mice more susceptible than C57BL/6 mice to experimental aerosol infection with Mycobacterium tuberculosis.. Infect Immun.

[36] Kindler V, Sappino AP, Grau GE, Piguet PF, Vassalli P (1989). The inducing role of tumor necrosis factor in the development of bactericidal granulomas during BCG infection.. Cell.

[37] Roach DR, Bean AG, Demangel C, France MP, Briscoe H (2002). TNF regulates chemokine induction essential for cell recruitment, granuloma formation, and clearance of mycobacterial infection.. J Immunol.

[38] Algood HM, Lin PL, Flynn JL (2005). Tumor necrosis factor and chemokine interactions in the formation and maintenance of granulomas in tuberculosis.. Clin Infect Dis.

[39] Saunders BM, Britton WJ (2007). Life and death in the granuloma: immunopathology of tuberculosis.. Immunol Cell Biol.

[40] Buchanan R (1918). Life phases in a bacterial culture.. The Journal of Infectious Diseases.

[41] Shleeva MO, Bagramyan K, Telkov MV, Mukamolova GV, Young M (2002). Formation and resuscitation of “non-culturable” cells of Rhodococcus rhodochrous and Mycobacterium tuberculosis in prolonged stationary phase.. Microbiology.

[42] Wayne L (1976). Dynamics of submerged growth of Mycobacterium tuberculosis under aerobic and microaerophilic conditions.. Am Rev Respir Dis.

[43] Barer M, Coates AR (2003). Physiological and molecular aspects of growth, non-growth, culturability and viability in bacteria.. Dormancy and low-growth states in microbial disease.

[44] Gomes M, Paul S, Moreira A, Appelberg R, Rabinovitch M (1999). Survival of Mycobacterium avium and Mycobacterium tuberculosis in acidified vacuoles of murine macrophages.. Infect Immun.

[45] Dannenberg AMJ (2006). Pathogenesis of human pulmonary tuberculosis.. Insights from the rabbit model.

[46] Cardona P (2007). New insights on the nature of latent tuberculosis infection and its treatment.. Inflamm Allergy Drug Targets.

[47] Morita RY, Kjelleberg S (1993). Bioavailability of energy and the starvation state.. Starvation in bacteria.

[48] Guillier L, Pardon P, Augustin J (2005). Influence of stress on individual lag time distributions of Listeria monocytogenes.. Appl Environ Microbiol.

[49] Prats C, Giró A, Ferrer J, López D, Vives-Rego J (2008). Analysis and IbM simulation of the stages in bacterial lag phase: basis for an updated definition.. J Theor Biol.

[50] Ulrichs T, Kaufmann S (2006). New insights into the function of granulomas in human tuberculosis.. J Pathol.

[51] Cardona PJ (2009). A dynamic reinfection hypothesis of latent tuberculosis infection.. Infection.

[52] Bui T, Dabdub D, George S (1998). Modeling bronchial circulation with application to soluble gas exchange: description and sensitivity analysis.. J Appl Physiol.

[53] Gil O, Guirado E, Gordillo S, Diaz J, Tapia G (2006). Intragranulomatous necrosis in lungs of mice infected by aerosol with Mycobacterium tuberculosis is related to bacterial load rather than to any one cytokine or T cell type.. Microbes Infect.

[54] Cardona PJ (2006). RUTI: a new chance to shorten the treatment of latent tuberculosis infection.. Tuberculosis (Edinb).

[55] Ayres JS, Schneider DS (2008). A signaling protease required for melanization in Drosophila affects resistance and tolerance of infections.. PLoS Biol.

[56] Gil O, Díaz I, Vilaplana C, Tapia G, Díaz J (2010). Granuloma encapsulation is a key factor for containing tuberculosis infection in minipigs.. PLoS One.

[57] Mollenkopf H, Kursar M, Kaufmann S (2004). Immune response to postprimary tuberculosis in mice: Mycobacterium tuberculosis and Miycobacterium bovis bacille Calmette-Guérin induce equal protection.. J Infect Dis.

[58] Sapoval B, Filoche M, Weibel E (2002). Smaller is better–but not too small: a physical scale for the design of the mammalian pulmonary acinus.. Proc Natl Acad Sci U S A.

[59] Caceres N, Tapia G, Ojanguren I, Altare F, Gil O (2009). Evolution of foamy macrophages in the pulmonary granulomas of experimental tuberculosis models.. Tuberculosis (Edinb).

[60] Gordillo S, Guirado E, Gil O, Díaz J, Amat I (2006). Usefulness of acr expression for monitoring latent Mycobacterium tuberculosis bacilli in ‘in vitro’ and ‘in vivo’ experimental models.. Scand J Immunol.

[61] Davis J, Ramakrishnan L (2009). The role of the granuloma in expansion and dissemination of early tuberculous infection.. Cell.

[62] Guirado E, Amat I, Gil O, Diaz J, Arcos V (2006). Passive serum therapy with polyclonal antibodies against Mycobacterium tuberculosis protects against post-chemotherapy relapse of tuberculosis infection in SCID mice.. Microbes Infect.

[63] Glatman-Freedman A, Casadevall A (1998). Serum therapy for tuberculosis revisited: reappraisal of the role of antibody-mediated immunity against Mycobacterium tuberculosis.. Clin Microbiol Rev.

[64] Murray J (2010). The structure and function of the lung.. Int J Tuberc Lung Dis.

[65] Rhoades E, Frank A, Orme I (1997). Progression of chronic pulmonary tuberculosis in mice aerogenically infected with virulent Mycobacterium tuberculosis.. Tuber Lung Dis.

[66] Canetti G (1955). The tubercle bacillus in the pulmonary lesion of man.. Histobacteriology and its bearing on the therapy of pulmonary tuberculosis.

[67] Herzog EL, Brody AR, Colby TV, Mason R, Williams MC (2008). Knowns and unknowns of the alveolus.. Proc Am Thorac Soc.

[68] Lenaerts AJ, Hoff D, Aly S, Ehlers S, Andries K (2007). Location of persisting mycobacteria in a Guinea pig model of tuberculosis revealed by r207910.. Antimicrob Agents Chemother.

[69] Buddle BM, Skinner MA, Wedlock DN, de Lisle GW, Vordermeier HM (2005). Cattle as a model for development of vaccines against human tuberculosis.. Tuberculosis (Edinb).

[70] Capuano SV, Croix DA, Pawar S, Zinovik A, Myers A (2003). Experimental Mycobacterium tuberculosis infection of cynomolgus macaques closely resembles the various manifestations of human M. tuberculosis infection.. Infect Immun.

[71] Miller M, Wei S, Cahalan M, Parker I (2003). Autonomous T cell trafficking examined in vivo with intravital two-photon microscopy.. Proc Natl Acad Sci U S A.

[72] Kipnis A, Basaraba R, Turner J, Orme I (2003). Increased neutrophil influx but no impairment of protective immunity to tuberculosis in mice lacking the CD44 molecule.. J Leukoc Biol.

[73] Rhoades E, Cooper A, Orme I (1995). Chemokine response in mice infected with Mycobacterium tuberculosis.. Infect Immun.

[74] Rodrigues M, Barsante M, Alves C, Souza M, Ferreira A (2009). Apoptosis of macrophages during pulmonary Mycobacterium bovis infection: correlation with intracellular bacillary load and cytokine levels.. Immunology.

[75] Khader S, Rangel-Moreno J, Fountain J, Martino C, Reiley W (2009). In a murine tuberculosis model, the absence of homeostatic chemokines delays granuloma formation and protective immunity.. J Immunol.

[76] Silver R, Li Q, Ellner J (1998). Expression of virulence of Mycobacterium tuberculosis within human monocytes: virulence correlates with intracellular growth and induction of tumor necrosis factor alpha but not with evasion of lymphocyte-dependent monocyte effector functions.. Infect Immun.

[77] Paul S, Laochumroonvorapong P, Kaplan G (1996). Comparable growth of virulent and avirulent Mycobacterium tuberculosis in human macrophages in vitro.. J Infect Dis.

[78] Cox R (2004). Quantitative relationships for specific growth rates and macromolecular compositions of Mycobacterium tuberculosis, Streptomyces coelicolor A3(2) and Escherichia coli B/r: an integrative theoretical approach.. Microbiology.

[79] Lee J, Remold H, Ieong M, Kornfeld H (2006). Macrophage apoptosis in response to high intracellular burden of Mycobacterium tuberculosis is mediated by a novel caspase-independent pathway.. J Immunol.

[80] Wallace JG (1961). The heat resistance of tubercle bacilli in the lungs of infected mice.. Am Rev Respir Dis.

[81] Munoz-Elias EJ, Timm J, Botha T, Chan WT, Gomez JE (2005). Replication dynamics of Mycobacterium tuberculosis in chronically infected mice.. Infect Immun.

[82] DesJardin L, Kaufman T, Potts B, Kutzbach B, Yi H (2002). Mycobacterium tuberculosis-infected human macrophages exhibit enhanced cellular adhesion with increased expression of LFA-1 and ICAM-1 and reduced expression and/or function of complement receptors, FcgammaRII and the mannose receptor.. Microbiology.

