# Supplementary figures and images for: Mathematical Modeling of Tuberculosis Bacillary Counts and Cellular Populations in the Organs of Infected Mice
Source: PLoS One. 2010 Sep 23;5(9):e12985. doi: 10.1371/journal.pone.0012985 (PMC2944881; doi:10.1371/journal.pone.0012985)

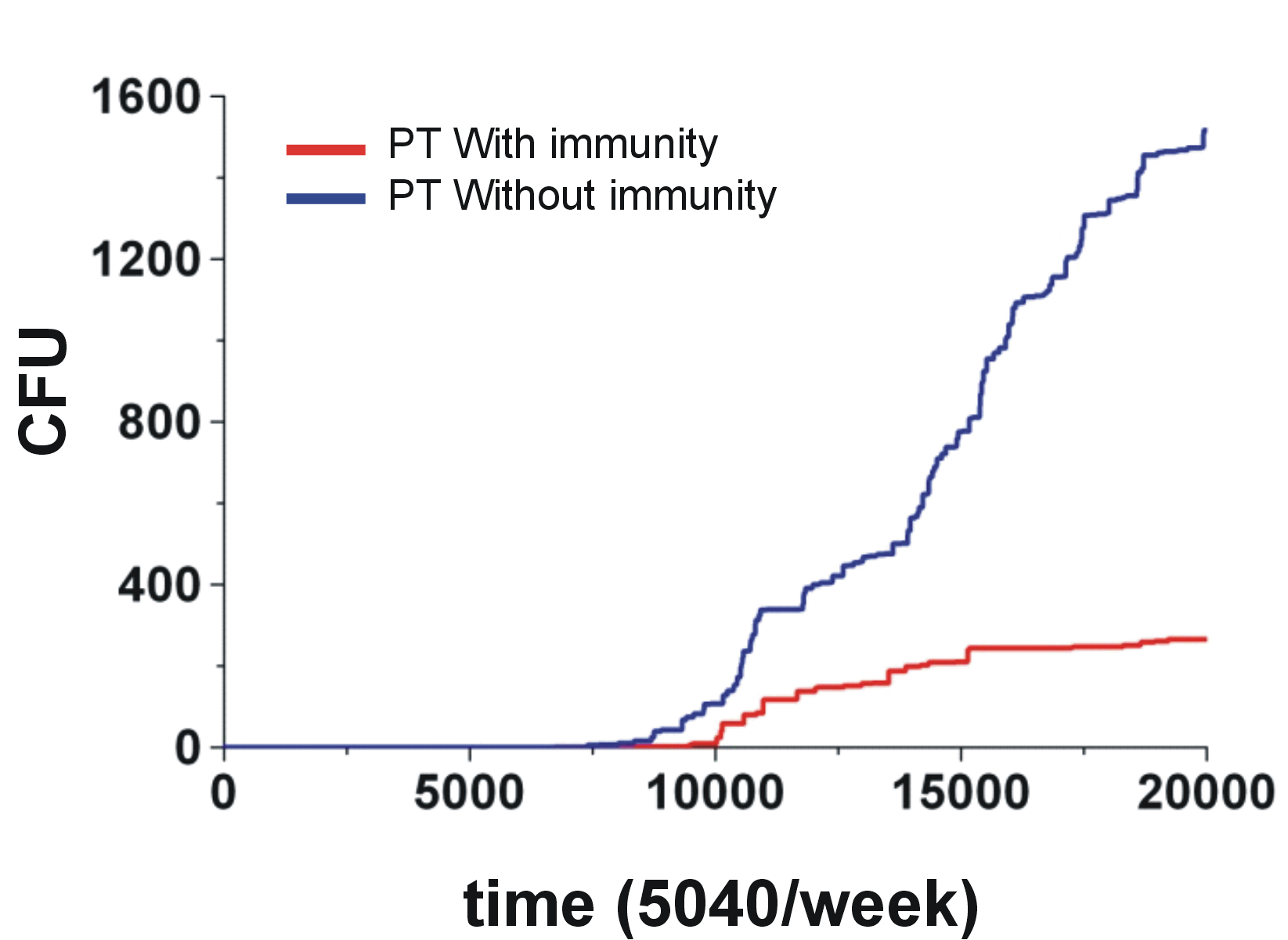

Supplement: Figure S1 — Evolution of the drainage of Foamy Macrophages (FM) in poorly tolerant (PT) hosts, according their immunity status (5.93 MB TIF) [file pone.0012985.s001.tif]

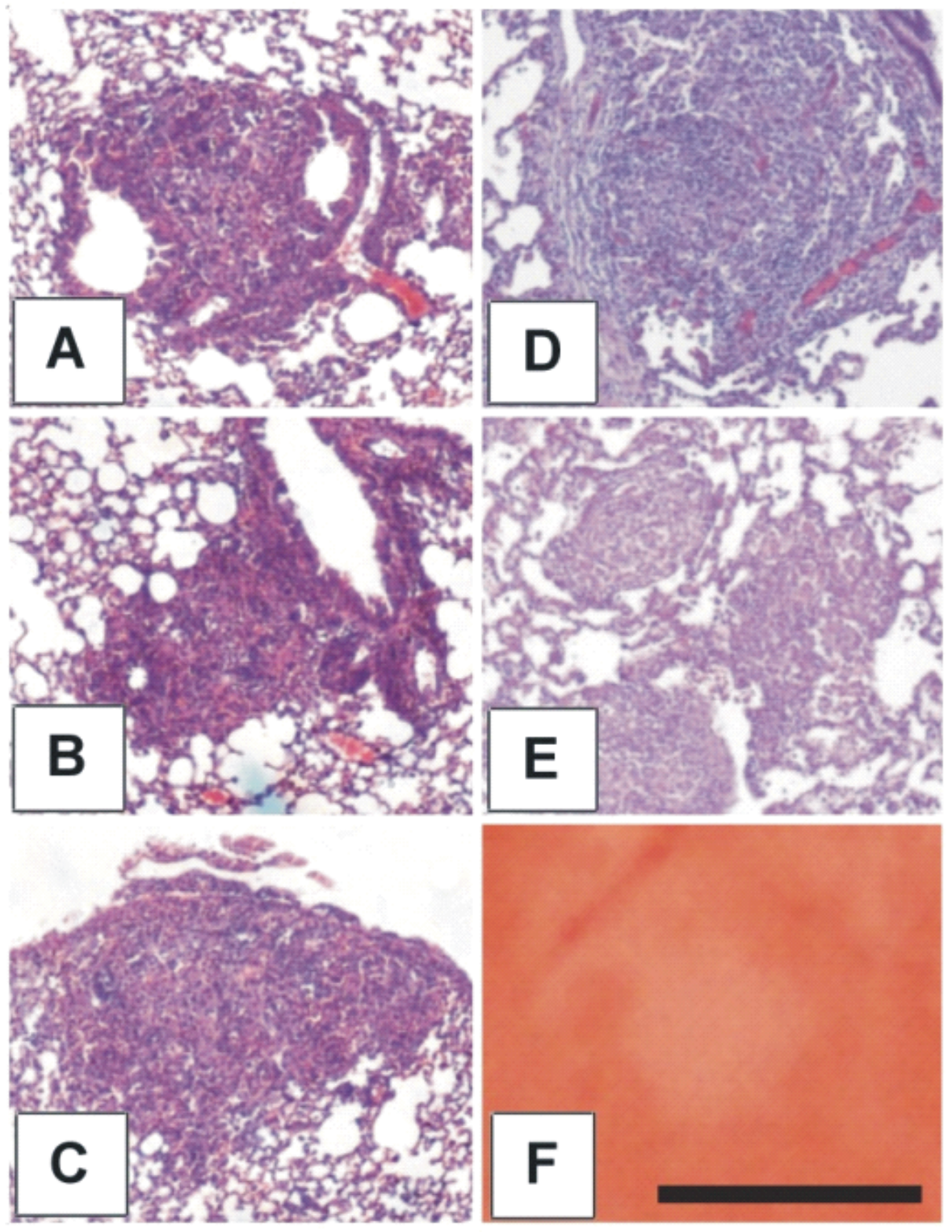

Supplement: Figure S2 — Photomicrographs of infected lungs in experimental models induced in mice (A to C) and mini-pigs (D to F) at weeks 3 and 5 respectively. Cuts were stained with haematoxylin-eosin (A to E) or visualized with a stereoscopic microscope, in the case of mini-pigs (F). (5.40 MB TIF) [file pone.0012985.s002.tif]

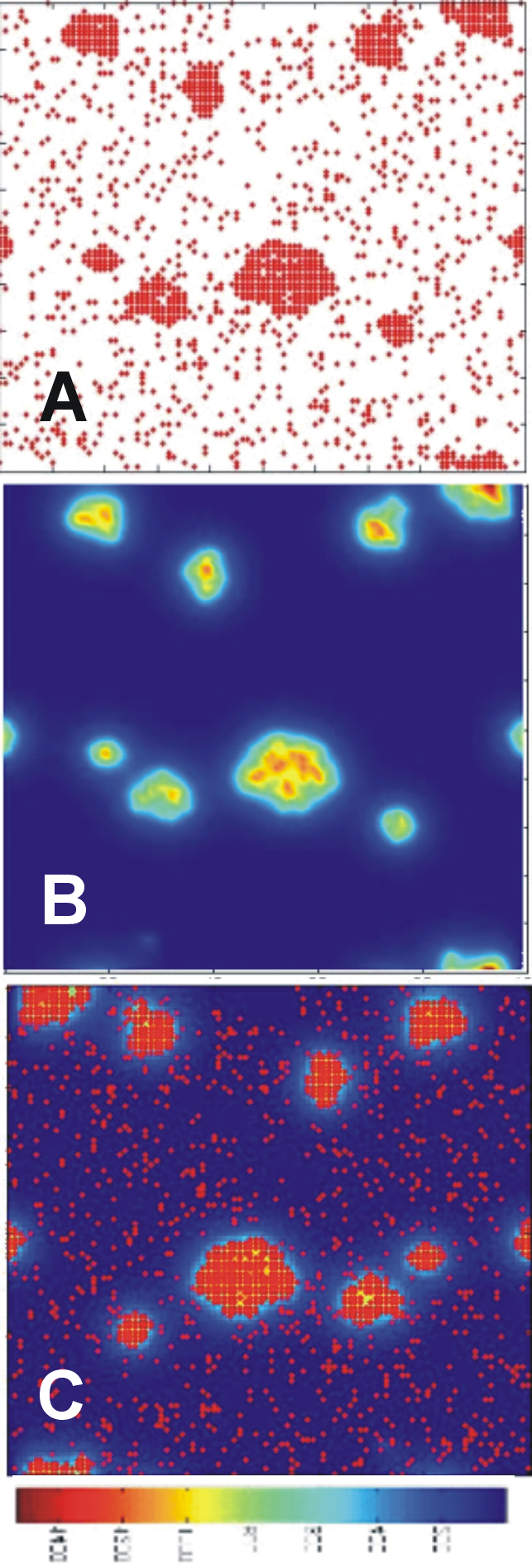

Supplement: Figure S3 — Macrophage infiltration and chemokine concentration in the space at week 4 post-infection in the case of a poorly tolerant host with an immune response. A: macrophage infiltration; B: chemokine production; C: combined image. (3.66 MB TIF) [file pone.0012985.s003.tif]

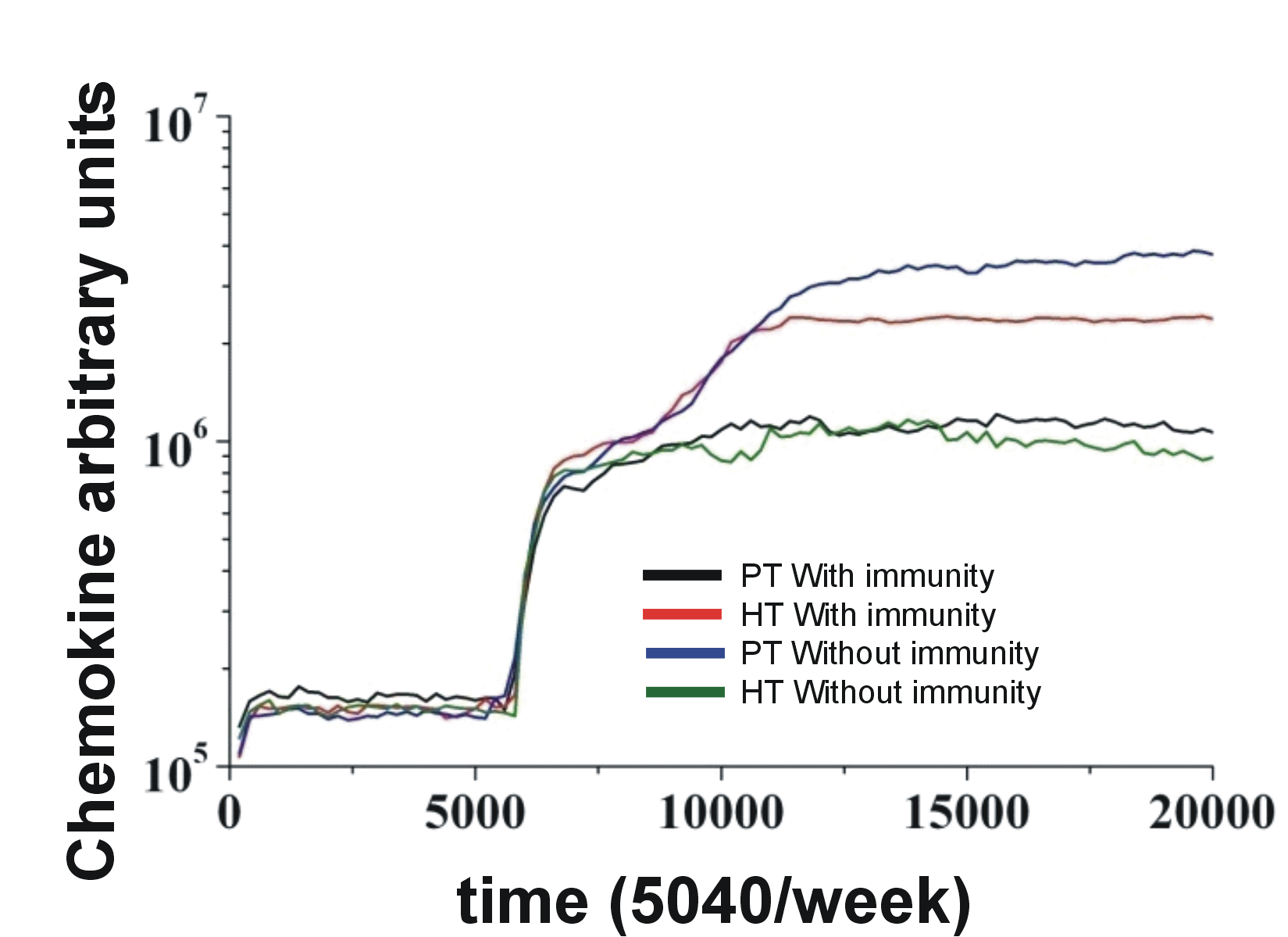

Supplement: Figure S4 — Evolution of the total amount of chemokines with time in all the cases studied. (5.93 MB TIF) [file pone.0012985.s004.tif]

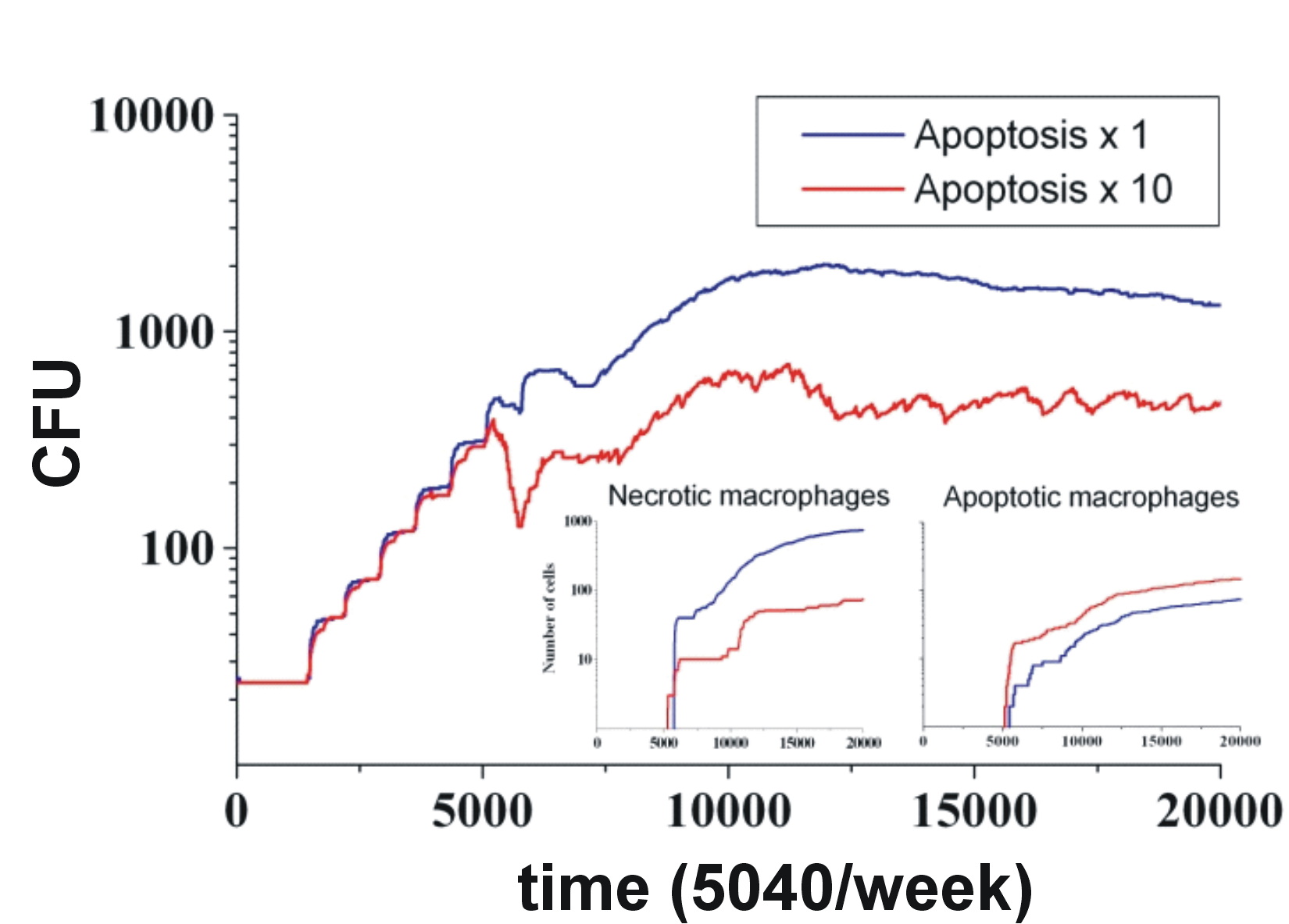

Supplement: Figure S5 — Influence of the apoptosis ratio on the bacillary concentration. The usual probability is compared with a 10-fold increase in the possibility of apoptosis, including the evolution of the numbers of necrotic and apoptotic macrophages in both cases (small squares). (5.86 MB TIF) [file pone.0012985.s005.tif]

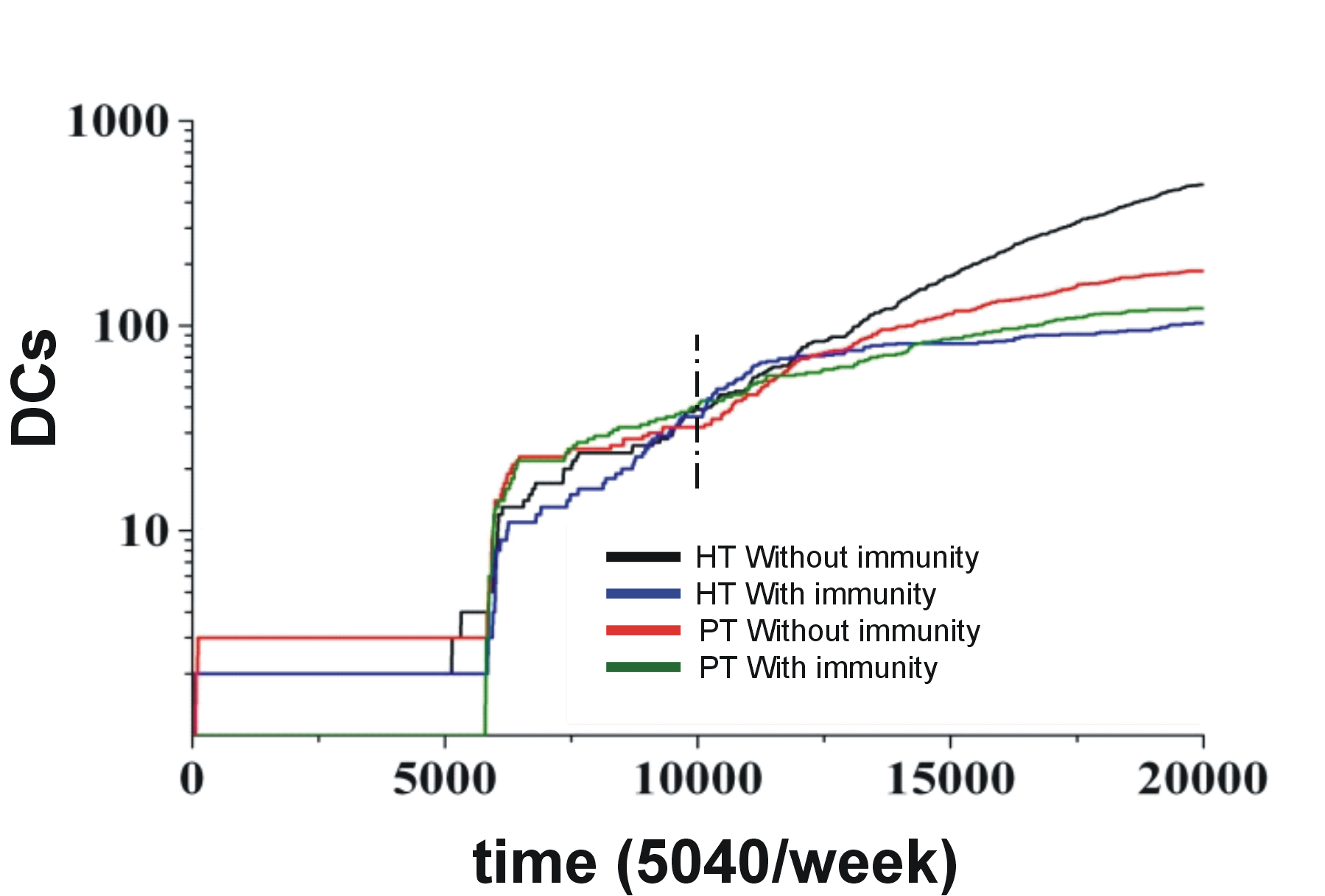

Supplement: Figure S6 — Evolution of dendritic cell (DC) formation in all the cases studied. (6.57 MB TIF) [file pone.0012985.s006.tif]

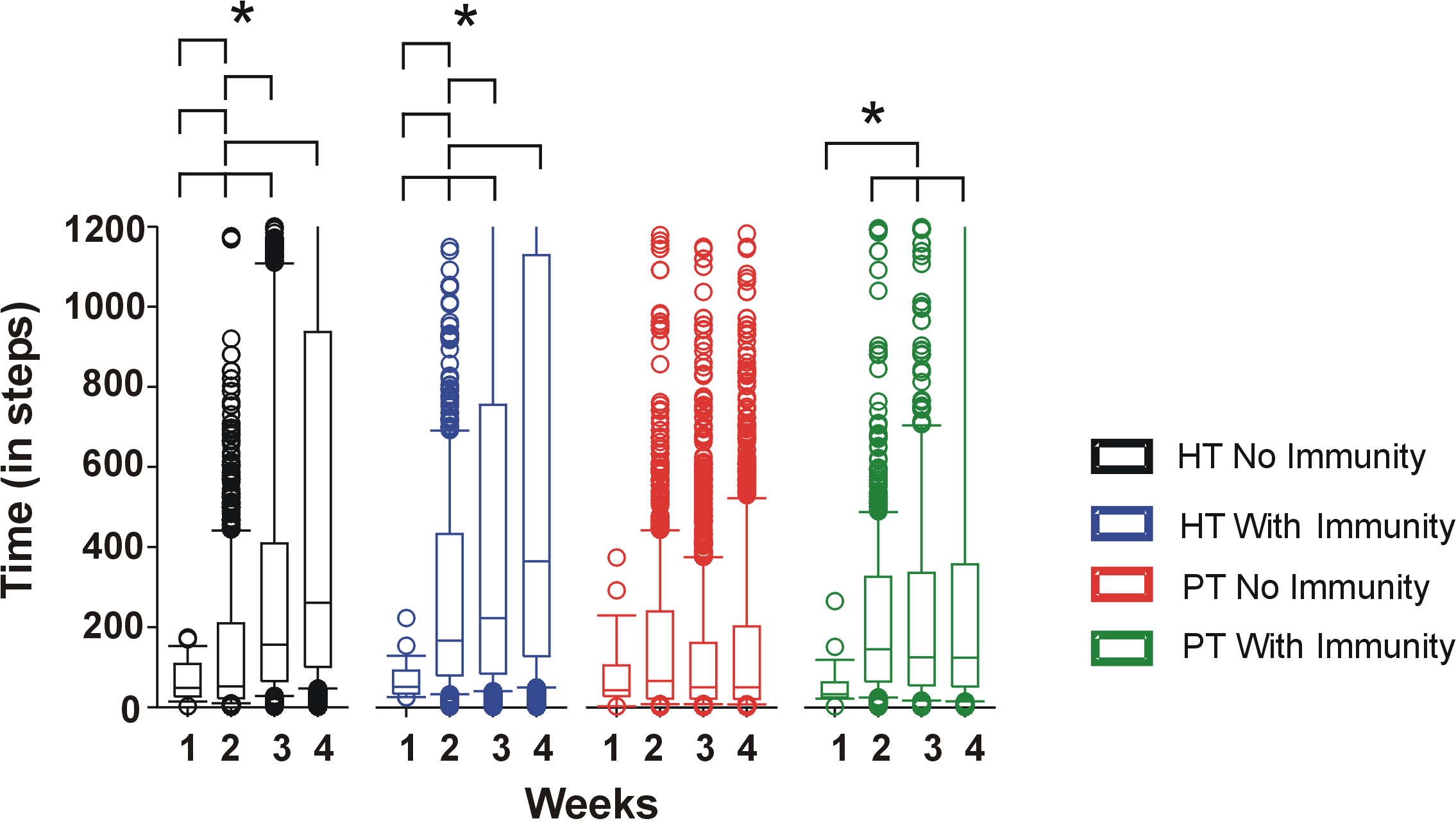

Supplement: Figure S7 — Distribution of Tlag for the four cases studied. Data are presented as boxes showing the 25th and 75th percentiles, and the 10th and 90th percentiles with error bars. The median is shown as a horizontal line inside the boxes. Differences between groups were determined using an all pairwise multiple comparison procedure (Dunn's Method), and are marked with * when significant. (8.72 MB TIF) [file pone.0012985.s007.tif]

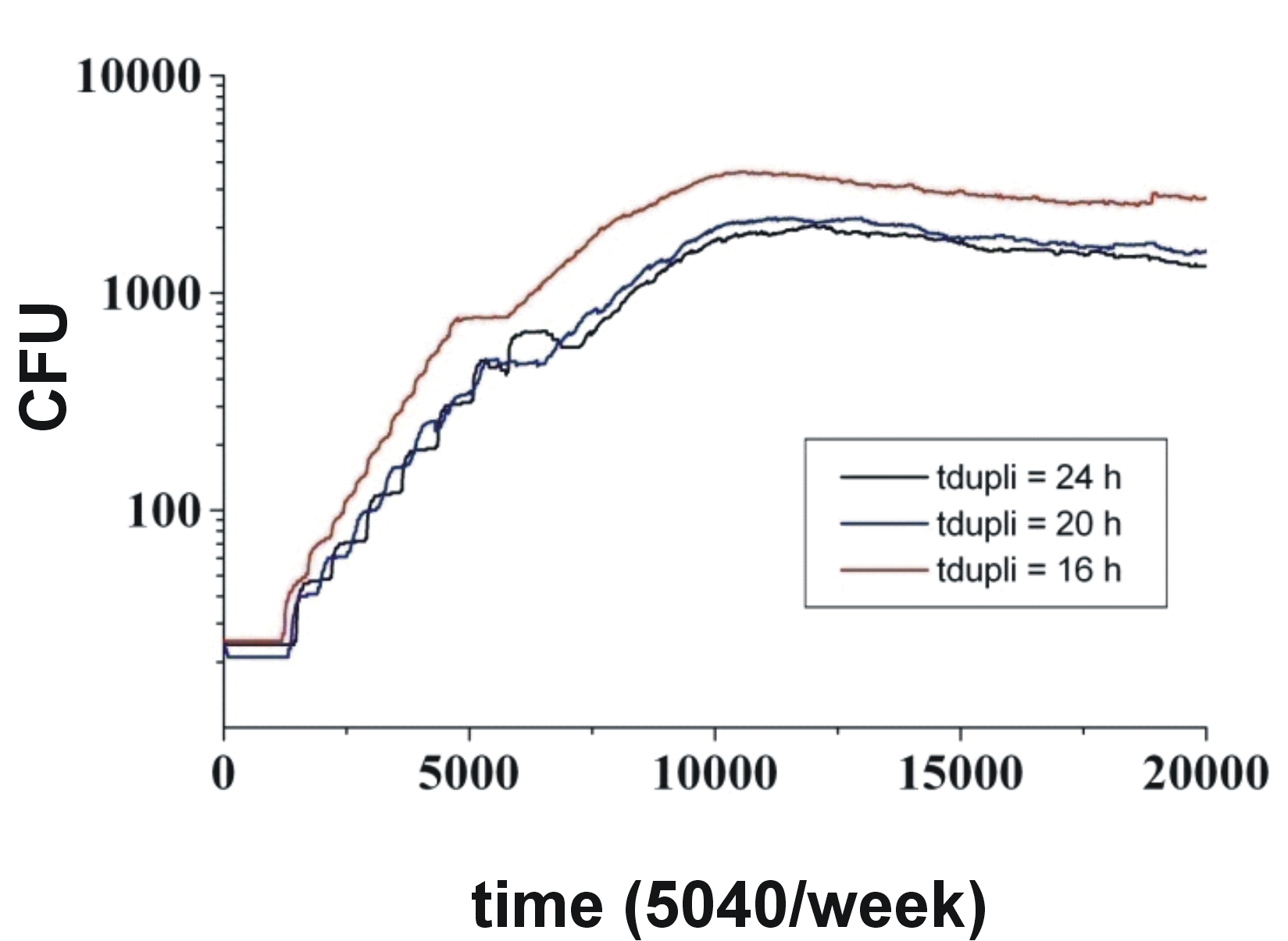

Supplement: Figure S8 — The influence of Tdupli and extracellular growth on the evolution of the total bacillary load, showing the influence of different Tdupli values. (5.96 MB TIF) [file pone.0012985.s008.tif]

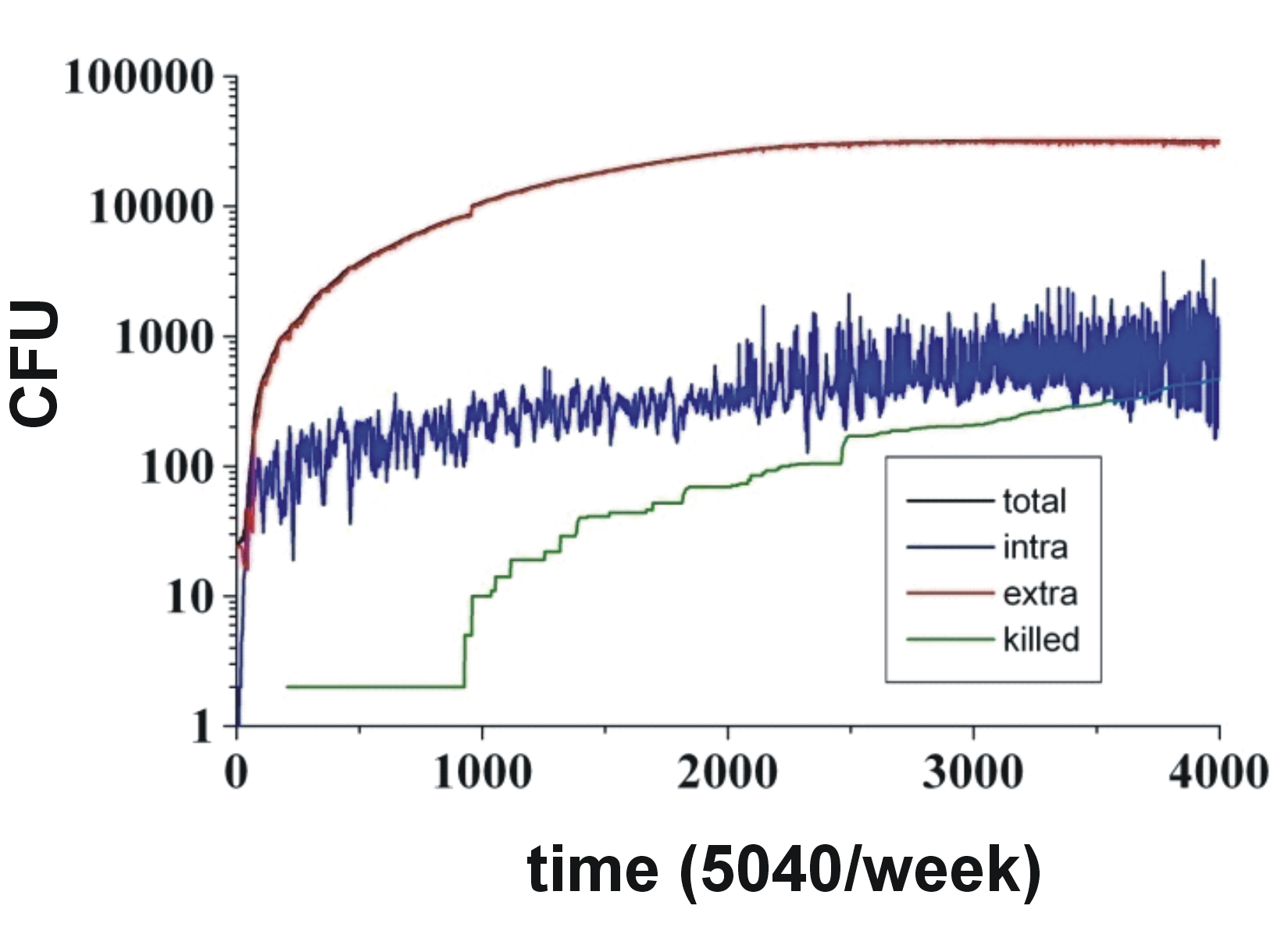

Supplement: Figure S9 — Recreation of a bacillary "chimera" with Tdupli = 20 minutes. (5.88 MB TIF) [file pone.0012985.s009.tif]

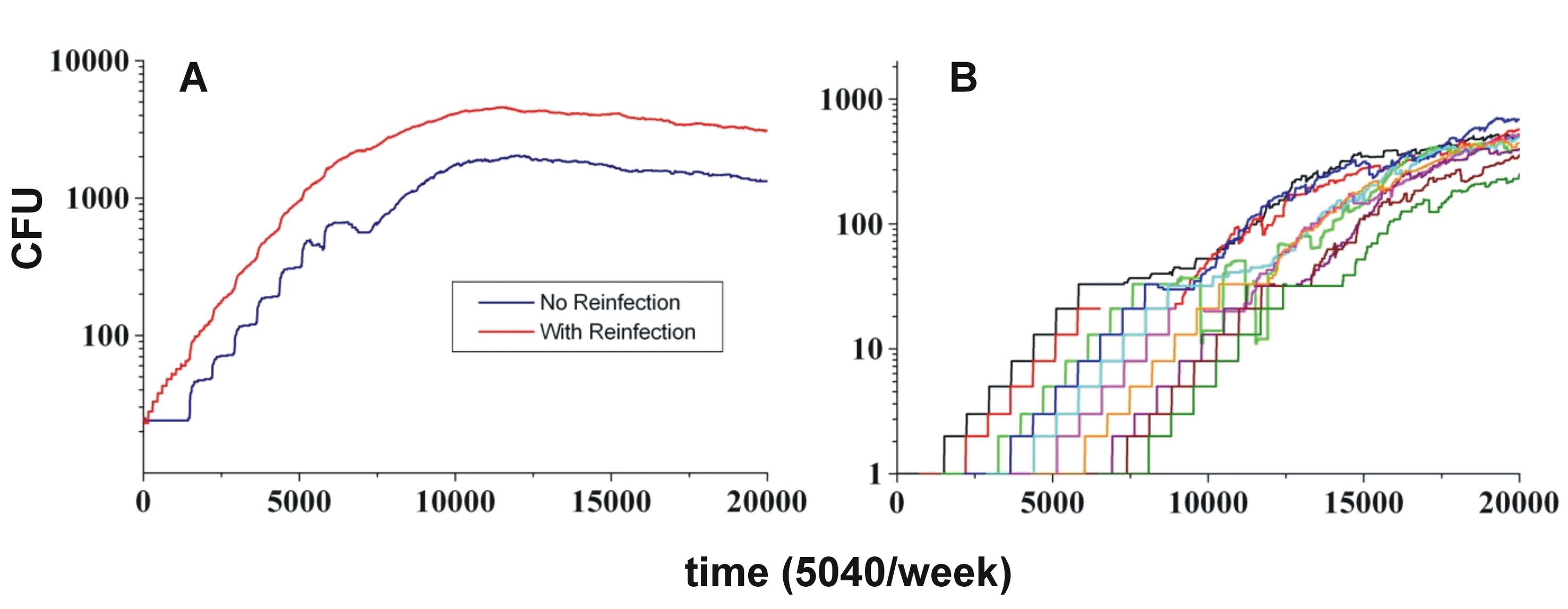

Supplement: Figure S10 — Role of reinfection in the evolution of the infection. Role of reinfection in the evolution of the infection. Picture A shows the standard inoculation in a PT host with immunity compared with the same host when constantly reinfected with 25 CFUs until t = 10,000 in the same lattice. Picture B shows the evolution of a whole lung of a person reinfected just 10 times with one bacillus in 10 different naïve lattices. The bacillary load is 5550. (10.17 MB TIF) [file pone.0012985.s010.tif]

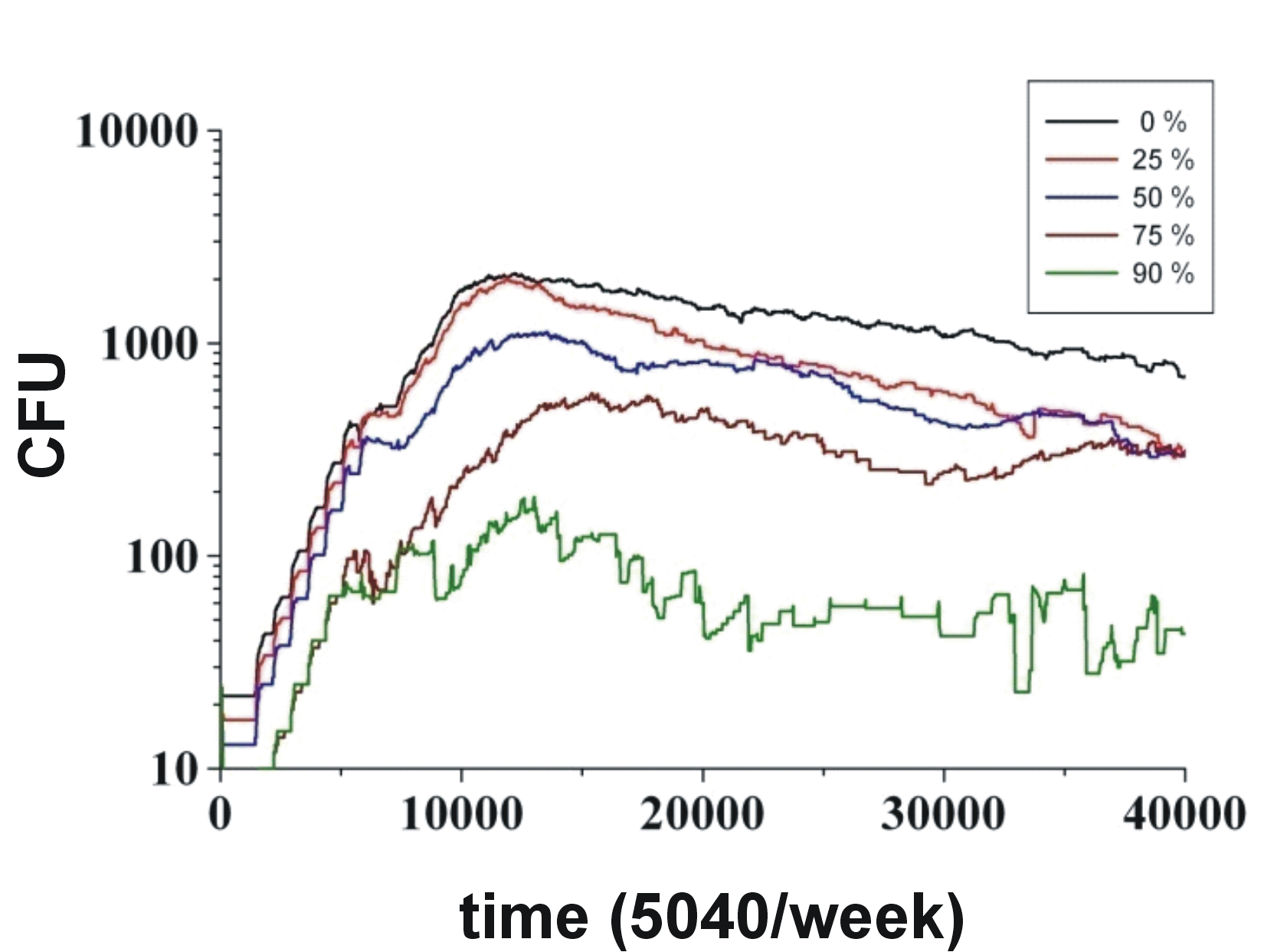

Supplement: Figure S11 — Presence of an hypothetical efficacious humoral response. Induction of a humoral response allowing the bacillus to be killed by any macrophage that phagocytes the opsonized bacillus, considering different percentages of activity (shown in the legend insert). (6.16 MB TIF) [file pone.0012985.s011.tif]

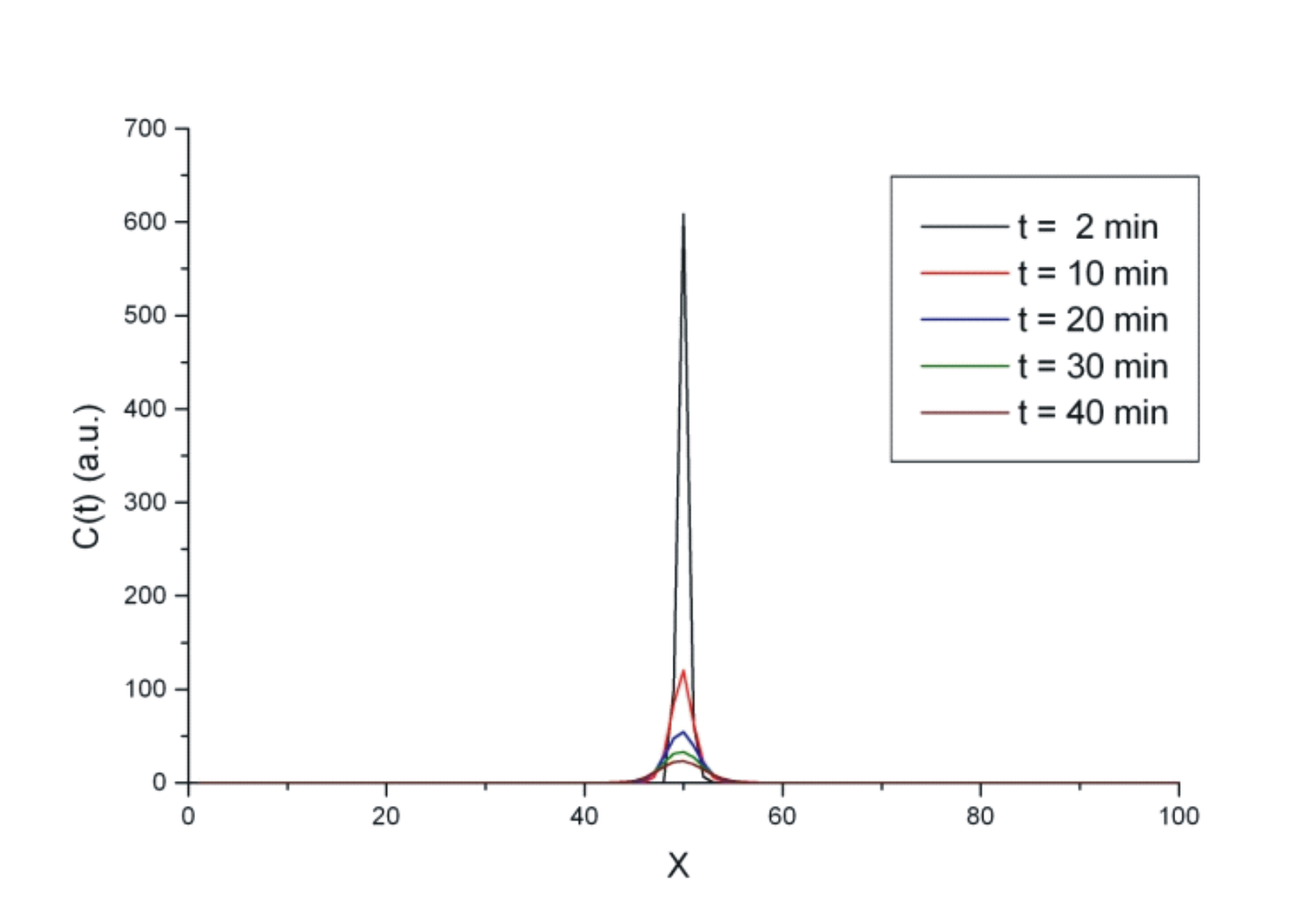

Supplement: Figure S12 — Dissemination of a single chemokine peak through space at different time points. (5.82 MB TIF) [file pone.0012985.s012.tif]
